# Repeated giant earthquakes on the Wairarapa fault, New Zealand, revealed by Lidar-based paleoseismology

**DOI:** 10.1038/s41598-020-59229-3

**Published:** 2020-02-07

**Authors:** Isabelle Manighetti, Clément Perrin, Yves Gaudemer, Stéphane Dominguez, Nicholas Stewart, Jacques Malavieille, Stéphane Garambois

**Affiliations:** 1 Université Côte d’Azur, OCA, IRD, CNRS, Géoazur, Valbonne Sophia Antipolis, France; 2 Institut de Physique du Globe de Paris, Sorbonne Paris Cité, Université Paris Diderot, CNRS, Paris, France; 3 Université de Montpellier, Géosciences Montpellier, CNRS, Montpellier, France; 4 Université Grenoble Alpes, Université Savoie Mont Blanc, CNRS, IRD, IFSTTAR, ISTerre, Grenoble, France

**Keywords:** Natural hazards, Solid Earth sciences

## Abstract

The Mw 7.8 2016 Kaikoura earthquake ruptured the Kekerengu-Needle fault resulting in the loading of its eastern continuation, the Wairarapa fault. Since the most recent earthquake on Wairarapa occurred in 1855 and is one of the strongest continental earthquakes ever observed, it is critical to assess the seismic potential of the Wairarapa fault, which might be prone to break. Using Lidar data, we examine its bare-earth morphology and reveal ~650 mostly undiscovered offset geomorphic markers. Using a code we developed in earlier work, we automatically measure the lateral and vertical offsets of these markers providing more than 7000 well constrained measurements. The data document the lateral and vertical slip profiles of the 1855 earthquake for the first time and show its total slip reached ~20 m at surface. Modeling the entire offset dataset reveals 7 prior earthquakes ruptured the entire fault, each similarly producing 16.9 ± 1.4 m dextral slip and ~0.6 m vertical slip at surface in the same central bend zone of the fault. Thus, the Wairarapa fault repeatedly produced giant earthquakes and is likely able to produce a similarly strong forthcoming event. The extreme large size of the Wairarapa earthquakes questions our understanding of earthquake physics.

## Introduction

A key to anticipate the size and damage potential of forthcoming earthquakes is to know the size (rupture length, displacement amplitude and distribution, magnitude) of previous large earthquakes on that fault^1^. The need to anticipate the size and damage potential of a forthcoming earthquake is especially critical when that fault is prone to break.

**Figure 1:**
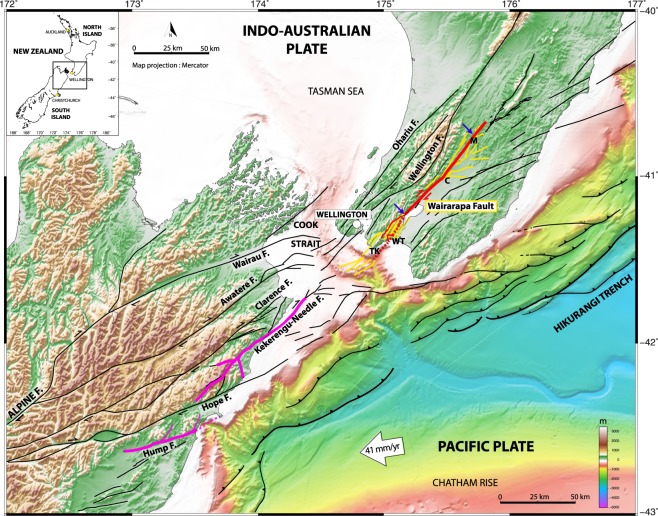
Tectonic setting of Wairarapa fault, New Zealand, and spatial relation to Kaikoura Mw 7.8 2016 rupture. Principal active faults in black and Wairarapa fault zone in yellow, with splay zones at both tips (see^9,14,32^ and New Zealand active fault database, https://data.gns.cri.nz/af), and 1855 Wairarapa rupture superimposed in red (length as inferred from present study). 2016 Kaikoura rupture in pink (from^10^ and others). Lidar stretch between blue arrows. TK: Turakirae, WT: Wharekauhau thrust (dotted), C: Carterton, M: Mauriceville. Figure done with GMT^56^.

This might be the case of the >100 km-long, fast-slipping Wairarapa fault (WP) (>1 cm/yr^2–5^), in the populated North Island of New Zealand (Fig. 1). WP is part of a group of major NE-trending strike-slip faults that dissect the southern and northern islands^2^. It extends right into the eastern continuation of the Hump-to-Needle fault system^6–9^ that ruptured in the Mw 7.8 2016 Kaikoura earthquake^10–12^. Although the transitioning of faulting in the Cook Strait is poorly understood^13^, it is likely that the two fault systems are kinematically linked^6,8,9,11,14–16^. Both the largest Kaikoura earthquake dextral slips (up to 12 m at surface^11,17^), and the abrupt rupture arrest occurred at the northeastern tip of the Needle fault system^18^, situated ~35 km away from the southern end of the WP fault^11^. The Kaikoura earthquake thus increased the stress loading on the southern WP^11,19–21^ as evidenced by the significant post-seismic deformation observed across the WP fault^20^. Together these may contribute to bringing the WP fault closer to failure. Furthermore, the WP fault most recently ruptured more than 160 years ago in 1855^22,23^, producing one of the strongest continental earthquakes ever observed (Mw 8.1–8.2). It broke the fault entirely (120–150 km^8,24,25^) and produced incredibly large lateral displacements at the ground surface, up to 17–18 m^26^. The 1855 earthquake thus had a dramatically large stress drop^26^. The 1855 earthquake initiated at the southern tip of the WP fault^22,27^. If a similar earthquake were to occur today, it would initiate where stresses have been most amplified by the 2016 Kaikoura earthquake.

The possibility of a forthcoming earthquake on the WP fault in the near future thus needs to be considered^11,21,28^). If the timing of such an event cannot be forecasted, its size may be by studying the past. Currently, little is known on the past earthquakes that broke the WP fault. The 1855 earthquake is the best known. However, only sparse measurements of the slips it produced at the ground surface have been published^2,4,26,29^ while its rupture length is debated^25^. Uplifted beach ridges in southern WP^30,31^ and trenching along the fault^5^ reveal that 4 large prior earthquakes occurred in the last ~6 kyrs^5,28^ with possibly up to 10 in the Holocene^24^. However, their slips and rupture lengths are poorly constrained.

The new data we provide here allow accurate estimations of the size (displacements, length) of the 1855 and 7 prior pre-historical earthquakes on the WP fault. We show that these past earthquakes were dramatically large. Beyond the high seismic hazard these large earthquakes pose in New Zealand, their extreme large size questions our understanding of fault and earthquake physics. Why do faults such as Wairarapa produce earthquakes much larger than commonly observed on other continental faults with similar length worldwide?

## Results

### Building a dense lateral and vertical fault offset database from Lidar data

WP is a strike-slip and reverse NE-trending, NW-dipping fault that extends over 120–140 km from offshore in the Cook Strait^6,14^ to Mauriceville^24,32^ (New Zealand active fault database, https://data.gns.cri.nz/af) (Fig. 1). We acquired airborne and terrestrial Lidar data to examine its bare-earth morphology at high resolution (horizontal spatial sampling ≤1 m, vertical precision 5–10 cm) (Methods, and Supplementary Fig. 1). The Lidar stretch extends over 70 km from the southern Wairarapa lake to Mauriceville, which does not include the thrust zone at southern WP (Wharekauhau and Turakirae zone^5^).

As recognized before (https://data.gns.cri.nz/af), the fault generally appears as a single strand, and forms a sharp, fairly linear and continuous trace highlighted by a few meters-high, east-facing scarp (Supplementary Fig. 1). More precisely, the fault is segmented into left-stepping segments of various lengths consistent with its dextral motion^33^ (Supplementary Fig. 1). Four major segments of similar length (20–30 km) are identified (A–H, I-NOw, NOe-T and U-Y, see Supplementary Fig. 1), separated by more pronounced steps or bends in the fault trace. The largest bend, ~20°, occurs at about the fault center. Secondary branches and long, oblique splay faults develop at the fault tips and in some places of its trace (Fig. 1 and Supplementary Fig. 1). The longest splays have developed in the central bend zone of the fault.

**Figure 2:**
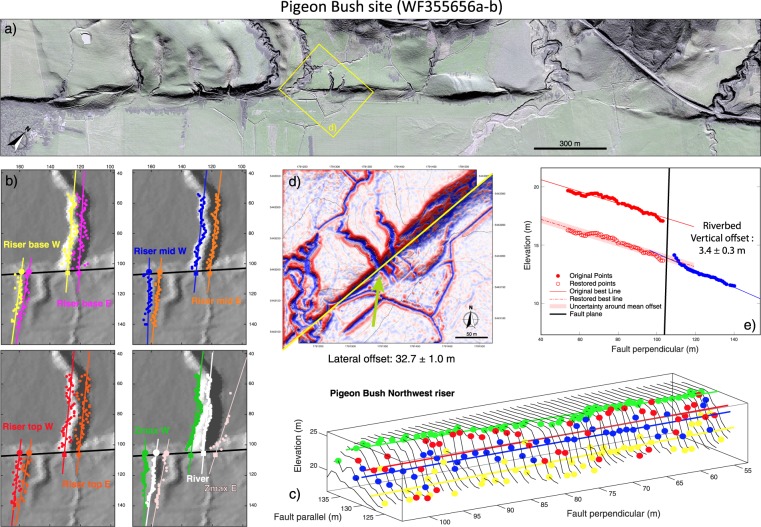
Major steps of automatic offset measurements with 3D-fault-offset code^34^ (see Methods for more details). (**a**) Lidar data in Pigeon Bush area (site with prior field measures, see Supplementary Spreadsheet); (**b**) automatic identification of 9 point clouds in each offset marker section on either side of the fault, and measurement of lateral and vertical offsets between the 9 pairs of best-fit lines through the point clouds (measurements in Supplementary Spreadsheet); (**c**) close-up view of points identified across northwest edge of channel and corresponding best-fit lines; (**d**) horizontal back-slip reconstruction of the targeted channel with the derived PREF lateral offset; (**e**) vertical back-slip reconstruction of the channel bed with the derived riverbed vertical offset. All figures done with GMT (^56^), and MATLAB (Matlab 2019, version 9.7.0 (R2019b). Natick, Massachusetts: the MathWorks Inc.).

The Lidar data reveals 643 offset geomorphic markers evenly distributed along the fault trace (Fig. 2 and Supplementary Fig. 1), 76% are stream channels of various sizes and 20% abandoned alluvial terrace risers. Most of those markers were previously unknown due to dense vegetation masking their trace in classical optical images and in the field. In fact, only 26 of these markers have been reported in the literature, along with the field measurements of their fault offsets^2,4,26,29^ (Supplementary Spreadsheet). Unpublished data (reported in^4^) suggest that up to ~100 more markers were identified in earlier field studies, but their precise location and offset measures are not available.

Here, we automatically measured the lateral and vertical offsets of the 643 markers, along both the main fault trace and the secondary faults, using the code “3D_Fault_Offsets” developed in an earlier work^34^ (Methods, Fig. 2 and Supplementary Figs. 2–3; measurements in Supplementary Spreadsheet). As we generally analyzed different sections of varying lengths per marker, and each series of measures includes 9 measurements of the lateral offset and 9 measurements of the vertical offset, we eventually produced 15 800 total offset measures. Of these, we only retained the most relevant and robustly constrained. The final database analyzed here includes 3567 lateral and 3567 vertical offset measures equaling more than 7000 offset measures. This is the largest offset database ever produced for any fault worldwide (see for instance^35–37^). This is also the first time that cumulative vertical offsets are measured systematically along a strike-slip fault, including the WP. For comparison, only 25 lateral and 20 vertical field offset measures have been published so far on WP^2,4,26,29^ (Supplementary Spreadsheet). Our automatic offset measures across the 26 markers identified on the field are consistent with the prior field measures (~70% consistency for both lateral and vertical offsets, see Supplementary Fig. 4A). More prior field measures seem to exist, possibly up to ~100, but they were not published nor located precisely^4^. We thus digitalized a graph synthesizing these measures (see their Fig. 4A in^4^; note that the graph only reports lateral offsets) to get a fair understanding of both their along-fault location and value, and to compare them to our measures. This comparison, shown in Supplementary Fig. 4B, demonstrates the consistency between these prior field and our automatic offset measures.

From our dense offset collection, we derived per marker (Supplementary Spreadsheet) a mean offset (noted OPT) automatically calculated by averaging all geomorphically-relevant offset measures (more than 3 measures for 85% of markers, more than 5 for half population, and up to 9–23) and a preferred offset (noted PREF), which we consider as the best value to reconstruct the pre-faulted marker geometry (Methods). The OPT and PREF offsets are similar within uncertainties in 96% (lateral) and 91% (vertical) of cases. The lower uncertainty-PREF offsets are thus used to provide a more accurate description of the offset data.

Of the entire dataset, 97% of the PREF and OPT lateral offsets are lower than 160 m, while 99% of the PREF vertical offsets are below 15 m (Supplementary Fig. 5). About 60% of the lateral offsets are greater than 18 m (maximum reported 1855 lateral slip^26^) demonstrating that the offset collection includes cumulative offsets that were accumulated from the addition of multiple past earthquake slips.

### Up to 8 large historical and pre-historical earthquakes revealed on WP fault

**Figure 3:**
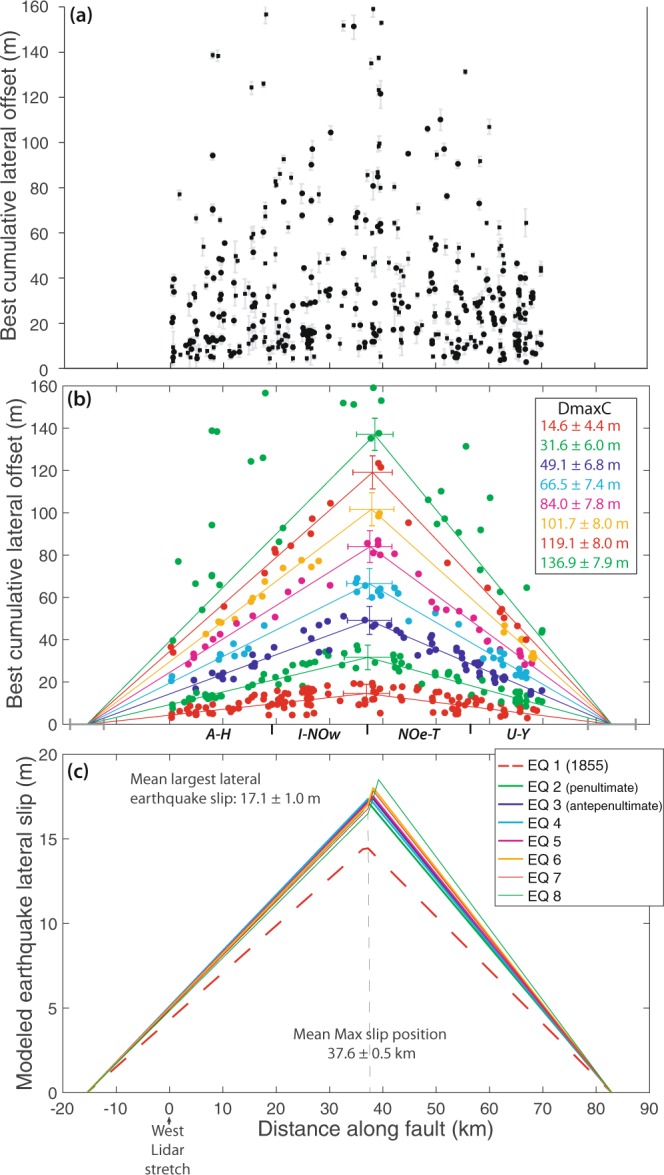
Modeling lateral offset data with Paleo_Slip-Length code to recover earthquake slips. (**a**) PREF lateral offsets of quality 1 and 2, in range 0–160 m, measured across main fault trace (data in Supplementary Spreadsheet); (**b**) Best model through the data (see Methods). Dmax C for cumulative maximum offset. Model parameters and uncertainties reported in Supplementary document A. Major fault segments are indicated (see Supplementary Fig. 1). The eighth earthquake is less well constrained as its function calculation includes a few outlier offset data; (**c**) Earthquake slip profiles derived from the model in (**b**), with mean largest slip and mean position of largest slip indicated. The 1855 slip is slightly lower than the other earthquake slips due to the larger variability of the preserved smallest offsets along the fault. It must be noted that the rupture length derived from the modeling is based on lateral offsets only. The rupture might have been longer if additional vertical slips occurred on the Wharekauhau thrust, as observed^5^.

Figure 3a presents the lateral offsets measured across the main fault. The complete PREF dataset and the OPT offsets are shown in Supplementary Figs. 6 and 7. Comprehensively, the lateral offset data fit into a rough triangle with an apex of ~140 m at 30–40 km along the fault. Within the triangle, offsets are not evenly distributed. There are zones void of data, and zones where data cluster. This suggests the existence of discrete offset subsets consistent with cumulative offsets building from the addition of discrete earthquake slips.

Lateral offsets lower than ~50 m form three dense, discrete clusters fairly continuous along the fault. The 3 clusters show a fairly symmetric triangular shape, with maximum lateral slip at 30–40 km along the fault, and slip decreasing progressively on either side of this zone. The lower cluster culminates at ~20 m, the second at ~35 m, and the third at ~50 m. Larger lateral offsets are more dispersed. However, two dense, discrete clusters are observed at 30–40 km along the fault, with lateral offsets of ~65 and 80 m. In the 30–40 km zone, larger offsets are less dense, yet form discrete groups at ~100, 120, 135 and 155 m.

The most recent 1855 earthquake on the WP fault produced 17 ± 1 m of maximum lateral displacement at the surface^26^ and broke the entire fault length. This suggests that the lower offset cluster, which represents the smallest lateral offsets preserved in the morphology, is the record of the 1855 earthquake. The triangular envelope shape of this offset record is consistent with the generic triangular pattern of earthquake slip distributions worldwide^38–40^ (see Supplementary Fig. 8). We interpret the second and third offset clusters as the cumulative records of the penultimate and the antepenultimate large earthquakes on the WP fault. Their triangular envelope shape is consistent with the generic triangular pattern of cumulative fault slip distributions^41,42^. The along-fault continuity of the two offset clusters suggests that both earthquakes broke the entire length of the WP fault similarly to the 1855 event. The discrete subsets of larger offsets in the 30–40 km zone might be the record of previous large earthquakes on the fault.

To recover the number and size of the past large earthquakes which built the measured cumulative offsets, we modeled the lateral offset data (Fig. 3b, Supplementary Figs. 9–12, and Supplementary document A). The code, “Paleo_Slip-Length”, is described in Methods (with Supplementary Fig. 13). It is based upon 3 hypotheses: (1) the measured offsets result from the addition of a variable number of discrete earthquake slips (a hypothesis commonly made in paleoseismology^36^); (2) the most recent earthquake slip profile (1855) and the earlier cumulative slip profiles have a triangular envelope shape, consistent with the generic form of both fault^41,42^ and earthquake^38–40^ slip profiles worldwide (see Supplementary Fig. 8); (3) all earthquakes have ruptured the same length of the WP fault (as suggested in earlier works^4^; we come back to this point in Discussion section). Using a Monte Carlo approach, the code thus searches for the triangular functions that best fit the entire lateral offset dataset. The final best models are derived from 50,000 iterations.

**Figure 4:**
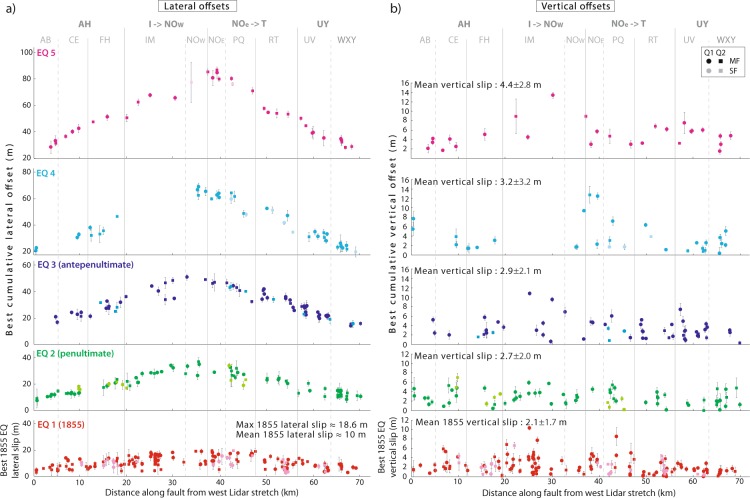
Lateral and vertical slip profiles of the 5 most recent earthquakes. (**a**) Lateral slip profiles. Profiles show the lateral offset data (PREF Q1&Q2, main and secondary faults) which have been assigned to one or other of the 5 lowest modeled functions (from Fig. 3b and Supplementary Fig. 11). Therefore, the first, lower profile in red is the 1855 earthquake lateral slip profile, while the above profiles are cumulative. The corresponding earthquakes are indicated. The vertical lines highlight the fault segments (from Supplementary Fig. 1) –the major ones indicated with thicker lines, which shape the lateral slip distributions, especially the 1855 one. The maximum and mean slips of the 1855 earthquake are indicated. (**b**) Vertical slip profiles. Vertical offsets are those measured across the markers whose lateral offsets are plotted in (**a**). Therefore, the first lower profile in red is the 1855 earthquake vertical slip profile, while the above profiles are cumulative. Earthquake numbering and vertical lines as in (**a**). The mean vertical slip of the 1855 earthquake is indicated along with the mean cumulative vertical slips of the prior earthquakes.

Figure 3b presents the best model for the cumulative lateral offsets of Fig. 3a, while Fig. 3c presents the 8 earthquake slip profiles derived from the model. While we describe the Fig. 3b model here, similar results were obtained from modeling all quality PREF and OPT lateral offsets (Supplementary Figs. 9–12 and document A). Figure 4a shows the coseismic and cumulative lateral slip profiles of the 1855 and 4 previous large earthquakes, respectively, inferred from the modeling (all profiles in Supplementary Fig. 17).

The three most recent large earthquakes are well defined. They likely ruptured the WP fault over ~100 km and produced a similar maximum lateral slip at the ground surface, of 16.4 ± 1.5 m located in the same zone of the fault (~37 km, Supplementary document A). Their lateral slip profile was similarly triangular and symmetric overall. However, the high density of offset data shows that the along-strike segmentation of the fault produced some persistent variability in the lateral slip distribution of the 3 (and up to 5) most recent large earthquakes (Fig. 4a), with moderate slip decrease and gentle slip gradients at the major inter-segment zones (thicker vertical lines in Fig. 4a), as commonly observed on earthquakes rupturing a segmented fault^43^. Four to five previous large earthquakes are partly recovered in the lateral offset data. They produced similar maximum slips of ~17.5 m in the same fault zone (37–38 km) as the 3 most recent earthquakes, and generated an overall triangular, symmetric lateral slip profile (Figs. 3b,c and 4, Supplementary Fig. 17 and document A). While our data suggest that the northern splay faults accommodated part of the earthquake slips (see measures on secondary faults in Supplementary Figs. 11–12 and Supplementary Spreadsheet), their individual contribution cannot be quantified.

The identification of the 8 earthquakes allows for the recovery of the vertical slips they produced for the first time. Figure 4b presents the coseismic and cumulative vertical slip profiles of the 1855 and 4 previous large earthquakes on the main fault, respectively (all profiles in Supplementary Fig. 17, and all data in Supplementary Fig. 18). The 1855 earthquake produced a variable vertical reverse slip averaging ~2 m with peak values of 6–8 m locally. The along-strike segmentation of the fault partly controlled the vertical slip variability. On average, the cumulative vertical slips increase with the earthquakes’ relative age (Supplementary Fig. 19) by about 0.6 m in each event (Fig. 4b and Supplementary Fig. 17). Like lateral slips, vertical slips seem larger in the central ~20–50 km part of the fault, and decrease on either side (Fig. 4b). Despite some variability, the vertical to lateral slip ratios average 0.11 ± 0.05 along the 8 earthquake slip profiles (highest 0.22 ± 0.16 ratio for 1855, Supplementary Figs. 20 and 21), yet with higher values at the rupture tips. The northward decrease of the vertical slips and the ~0.1 mean vertical to lateral slip ratio agree with the few prior estimates^4,22,24,26^.

With the lateral and vertical slips, we could measure the total slip profile of the 1855 earthquake for the first time as well as the cumulative total slip profiles of the previous earthquakes (Supplementary Figs. 20 and 21). Differently from previous estimates^26^, this reveals that the 1855 earthquake produced up to ~20 m of total slip at the ground surface with a mean slip of ~11 m.

## Discussion

Lidar data are confirmed to be invaluable in recovering the memory of past large earthquake slips on a fault^1,36,44^. Our study also confirms the robustness of the code 3D-fault-Offsets^34^ to automatically characterize the geometric properties of offset geomorphic markers in topographic data and measure their lateral and vertical offsets accurately. When these offset measures are dense, as is the case here, they form discrete clusters indicative of past earthquake slips and rupture lengths. The along-fault continuity of the three “lower”, best-constrained offset clusters (Fig. 3a) suggests that the three most recent earthquakes which produced them broke the entire length of the WP fault, which was indeed observed in the 1855 event. Based on this observation, we have hypothesized that all prior earthquakes identified in our offset data were similar, i.e., broke the WP fault entirely. One could argue, however, that some of these earthquakes were smaller and ruptured only a section of the fault including one, two or three of its four major segments (Supplementary Fig. 1; rupture length is controlled by source fault segmentation^45,46^). If this is the case, this smaller rupture length would range between ~20 and 60 km (see Fig. 4a). From the available earthquake slip-length scaling relations we infer that the lateral slip produced by such shorter ruptures would range between <1–3 m^47^ and 5–10 m^46^ (WP is an immature fault). Although the upper bound is large, it is still significantly lower than the mean slip increments systematically observed in the central part of the fault (15–17 m on average, Fig. 3). Furthermore, if the fault segments have ruptured in different earthquakes, strong slip gradients would be observed at their tips where earthquake slip dropped down to zero. However, we observe only gentle slip gradients at inter-segment zones displaying moderate slip decrease, but not slip arrest. Together these confirm that the past earthquakes identified in our offset data broke the entire length of the WP fault, and produced large lateral slips of ~16.5 m on average.

The large slips systematically produced by the WP earthquakes (16.5 ± 2.2 m of dextral slip on average and up to 20 m of total slip, Supplementary document A) make them the largest ever observed on a continental fault, especially since earthquake slips are generally greater at seismogenic depths^38^. Conversely, their rupture length was moderate, in effect, producing a very high earthquake slip to length ratio, and hence, stress drop^26,46^. The earthquakes that repeatedly ruptured the WP fault in the past were thus all extremely energetic and hence potentially very damaging. We attribute this strong seismic potential of the WP fault as being related to its structural immaturity^46^. The WP fault indeed initiated less than 1–3 Myrs ago^6,48^ and accommodated no more than 10 km of total lateral displacement^49^, which makes it an immature fault^46^. This is consistent with its fairly symmetric slip distribution^38,46^, and its highly segmented trace^25,33^. Immature faults have been shown to produce the largest stress-drop^46^ and strongest ground motion earthquakes^50^. This makes the WP fault one of the most threatening faults in New Zealand. If we assume that every large earthquake on the fault ruptured its entire length (120 ± 20 km) and width (25–45 km^51,52^) and produced ~20 m of total displacement and ~11 m of mean slip (minimum estimates since slips are generally greater at depth), we infer that these earthquakes had a minimum moment magnitude of 7.9–8.2^53^.

The hazard posed by the WP fault is amplified by three factors. Firstly, as said earlier, the 2016 Kaikoura earthquake loaded the southern part of the fault, which may contribute to advancing the expected timing of its forthcoming rupture^21^. Secondly, the 1855 earthquake and likely the 4 earlier large events nucleated at the southern tip of the fault, on a secondary reverse fault zone (Wharekauhau^5,8,22,30^, Fig. 1). This region of apparently systematic nucleation is where stresses have been most amplified by the Kaikoura earthquake. Furthermore, the rupture of the reverse fault(s) systematically produced large vertical slips (which raised beach ridges by ~5 m in each earthquake^5,31^), which in turn induced a several meters-high tsunami^22^. Finally, while large earthquakes on the WP fault do not seem to cluster in time (long recurrence intervals separate them, 1230 ± 190 yrs^5^), they might cluster spatially, as observed in other fault systems worldwide^54^. In fact, the nearby 1848 Awatere large earthquake in the southern island preceded the 1855 WP earthquake by 7 years. Furthermore, both earthquakes triggered the rupture of distant faults on the opposite side of the Cook Strait^8^. In the last ~1000 yrs, 2 large pre-historical earthquakes on the Kekerengu fault in the southern island were closely followed by earthquakes on the WP fault^11^. Together these spatial sequences suggest that the Wairarapa fault is interacting with at least the Kekerengu, Needles and Awatere faults in the southern island^14^ (Fig. 1), altogether forming a kinematically connected seismogenic fault zone across the Cook Strait. The 2016 rupture of the Hump-to-Needle fault might thus have the potential to trigger a forthcoming clustered earthquake on the WP fault. From our results, we anticipate that the largest slips may occur in the central bend zone of the fault (at 37.9 ± 0.5 km from west Lidar stretch, Supplementary document A), near Carterton, and decrease progressively on either side up to the fault tips.

Our dense offset database provides a robust basis to identify the geomorphic markers most appropriate to date the identified earthquakes. Along with the measures of the largest and mean WP earthquake slips we provide here, this dating should help estimating the probability of rupture of the WP fault over the next 100 yrs^7,28,55^.

The WP fault produces earthquakes much stronger than commonly observed on continental faults of similar length. It has been suggested that such large stress drop earthquakes are primarily produced by immature faults^46^, i.e., faults with short slip history generally less than a few Myrs. The WP fault is indeed immature^6,48,49^. Following our previous work^40^, we argue that, while immature faults or fault sections are embedded in less compliant damage zones than are mature faults, they have a higher strength and a higher fracture energy due to their greater segmentation (i.e., greater density of strong inter-segment zones, see Fig. 7 in^40^) and a higher density of protrusions and contacts needed to be broken. The Wairarapa fault is a natural laboratory where these hypotheses could be examined to better understand and characterize the physical properties of an immature fault and their impacts on the earthquake process. These analyses would allow a comparison with the mature Alpine fault nearby, which is currently undergoing drilling (see DFDP project and related publications).

## Methods

### Lidar data acquisition and processing

We acquired the LiDAR data in the framework of the project CENTURISK funded by the French Research Agency (ANR, CENTURISK Risknat09-456076, PI: I. Manighetti) (Supplementary Fig. 1).

Airborne LiDAR and digital imagery was collected between 8-9 April 2012, using NZ Aerial Mapping’s Optech ALTM 3100EA LiDAR system and Trimble AIC medium format digital camera. The data was collected flying 900 meters above the ground, and using a field of view of 20 degrees either side of nadir. The system PRF was set at 70 kHz. The LINZ geodetic reference mark KB79 and a mark that NZAM established at Masterton airfield were used for the collection of GPS receiver station data during the aerial data acquisition. The LiDAR sensor positioning and orientation (POS) was determined using the collected GPS/IMU datasets and Applanix POSPac software. This work was all undertaken in NZGD2000 coordinate system using the data collected at the geodetic reference mark for the DGPS processing. The POS data was combined with the LiDAR range files and used to generate LiDAR point clouds in New Zealand Transverse Mercator (NZTM) map projection but NZGD2000 ellipsoidal heights. This process was undertaken using Optech LMS LiDAR processing software. The data was checked for completeness of coverage. The relative fit of data in the overlap between strips was also checked. The point cloud data was then classified into ground, first and, intermediate returns using automated routines tailored to the project landcover and terrain. These, and subsequent steps were undertaken using TerraSolid LiDAR processing software modules TerraScan, TerraPhoto and TerraModeler. The Trimble camera images were developed into 8 bit per channel uncompressed TIFF format images. The LiDAR POS data was transformed for use with the camera, and this data was used with the automated classified ground LiDAR point cloud data to produce orthophotos with a ground sample distance of 0.2 m. Comprehensive manual editing of the LiDAR point cloud data was undertaken to increase the quality of the automatically classified ground point dataset. This editing involved visually checking over the data and changing the classification of points into and out of the ground point dataset. Attention was particularly focused on areas of vegetation. The Trimble orthophotos were used as a backdrop when undertaking the manual editing. The height and positional accuracy of the LiDAR data was checked by comparing it to overlapping LiDAR surveys that NZAM had conducted earlier at small locations within the project areas. The new data was found to fit well with the existing data.

To validate the efficacy of the airborne LiDAR to produce robust offset measurements even for smaller lateral offsets (i.e. < 10 m), we conducted a field campaign in April 2016 to acquire terrestrial LiDAR data (TLS) at three sites along the WP fault. We used a FARO Laser Scanner Focus 3D X 350 belonging to the laboratory Géoscience Montpellier. The scanner is a high-speed three-dimensional laser scanner. It can acquire >500,000 points/s from a distance of 0.6 m up to 300 m. This large amount of points translates to a very high point density, enabling the generation of very-high resolution DEMs (e.g., averaged spatial resolution ≤20 cm, measurement accuracy ≤2 mm). We acquired TLS measurements at 3 sites (location in Supplementary Fig. 1). Site F consists of a series of offset river terraces and ancient channels formed by the adjacent Waiohine River, which have been studied in earlier works^29^ (Supplementary Spreadsheet), allowing comparison with prior measurements. We made a total of 17 scans. Sites I West and I East consist of a series of offset beheaded channels, which have not been documented before. We measured a total of 47 scans to cover the entire Site I.

### Automatic offset measurements

We automatically measured the lateral and vertical offsets of the  >640 markers using the Matlab code “3D_Fault_Offsets” our group developed recently^34^ (code available in^34^; Fig. 2 and Supplementary Figs. 2-3). The code is dedicated to analyze sub-linear geomorphic markers in topographic data. It works on a case-by-case analysis. In a topographic data subset enclosing a given offset marker, the user traces the fault line manually and draws two rough polygons enclosing the two marker sections. The fault dip is taken into account. The code then mathematically and automatically identifies in each polygon up to nine of the possible geometric characteristics per marker: the riverbed, identified as the zone of lowest elevation (Min Z referred to as “river”, one point cloud); riser or scarp base and top (referred to as “bot” and “top”), identified through their slope break using the maximum (i.e. maximum convexity of slope) and minimum Laplacian (i.e. maximum concavity of slope) of the topography, respectively (four point clouds on either side of a riverbed); riser or scarp steepest central part or “free face” (referred to as “mid”), identified through the measurement of the maximum gradient of the topography (two point clouds on either side of a riverbed); and the ridge or crest identified as the zone of maximum elevation (referred to as “Max Z”; two point clouds on either side of a riverbed). The code searches for these 9 specific features systematically along fault parallel topographic profiles that cover the entirety of the polygon. This eventually populates each polygon with nine individual point clouds on either side of the fault. Using the least-squares method, the code then computes a 3-D linear regression through each of the nine point clouds on either side of the fault, in effect creating 18 lines of best-fit. After this first regression, the code automatically removes the artifact points related to the polygon edges and the obvious outliers of the lines (interquartile method). It then recalculates the best-fit lines from these “cleaned” point clouds. The eventual best-fit lines characterize the marker geometry in 3D. Note that some of the best-fit lines might not be well constrained or geomorphically relevant. If so, the user can remove them at a later stage. Each 3D line of best-fit intersects the dipping fault plane creating a piercing point whose x, y, and z coordinates are recorded. Based upon the common assumption that paired piercing points on either side of the fault were initially the same “pre-faulted” point, the code computes the 3 components of the slip vector that joins the paired piercing points. It specifically calculates the horizontal and vertical offsets by subtracting the x and z coordinates of the corresponding piercing points on either side of the fault. The 18 offset calculations (nine lateral and nine vertical offset measures per marker) are done systematically regardless of their geomorphic relevance, which can be defined subsequently. Through a Monte Carlo approach, the code calculates the total uncertainty on each offset. Eventually, 3D_Fault_Offsets reconstructs the pre-faulted marker geometry in the horizontal and vertical planes (Fig. 2 and Supplementary Figs. 2-3).

The code is entirely automatic and we used it with no manual intervention (but fault and polygon tracing). The 9 lateral and the 9 vertical offset measures obtained per marker provide a unique opportunity to examine the variability of the offsets across the entirety of the marker. The code uses probability density functions (PDF) to extract the most robust offset values and their uncertainties. These uncertainties represent the largest possible errors on the offsets for they integrate the full range of offset variability.

We used a constant 80 ± 10° NW dip for the analyzed faults. We generally used different polygon sizes per marker to best capture their geometry, and therefore performed several series of measures per marker (Supplementary Spreadsheet). To calculate the mean offsets per marker (OPT), we only retained the geomorphically meaningful offset values (explanation in^34^). Because they integrate the full range of offset variability, they generally have fairly large, conservative uncertainties (generally 0–30% of the offset for lateral offsets, Supplementary Fig. 5b). Note that the OPT offsets have no equivalent in literature. The PREF offsets are the offset value among the 9 or more which we consider as the best value to reconstruct the pre-faulted marker geometry. We defined them through careful examination of all possible horizontal back-slip reconstructions. As they represent a single measurement out of the many possible per marker, they are equivalent to the single offset measurements provided per marker in literature. As expected, the PREF offsets have lower uncertainties (generally 0–20% and 0–30% of the offset for lateral and vertical offsets, respectively, Supplementary Fig. 5b) than the OPT offsets, comparable to ranges on prior measures in literature.

We qualified the robustness of the PREF and OPT offsets by assigning them a quality 1 (very good), 2 (good), or 3 (poor) weight based on the preservation of the marker and the ability of the lateral offset to reconstruct its pre-faulted geometry and that of neighboring features (Supplementary Spreadsheet and Supplementary Figs. 2–3).

### Offset modeling

We wrote a Matlab code, “Paleo_Slip-Length”, to model the cumulative lateral offset data and recover the individual earthquake slips that accumulated over time to build them (Matlab 2019, version 9.7.0 (R2019b), Natick, Massachusetts: the MathWorks Inc.). The code is based upon 3 hypotheses: (1) the measured offsets result from the addition of a variable number of discrete earthquake slips (hypothesis commonly made in paleoseismology^36^); (2) the most recent earthquake slip profile (1855) and the earlier cumulative slip profiles have a triangular envelope shape, consistent with the generic form of both fault^41,42^ and earthquake^38–40^ slip profiles worldwide; (3) all earthquakes have ruptured the same length of the WP fault (as suggested in earlier works^4^).

The code is made of three connected routines: innermost, intermediate, and outermost.

The innermost routine reads the data (L, D, W) where D and L are the measured displacement and its along-fault position, respectively, and W the weight on the offset. This weight is calculated with a separate routine; the user can define it as depending on the offset uncertainty (W = 1/sigma^2), on the quality weight (weights assigned to 1, 0.75, and 0.5 for Q1, Q2 and Q3, respectively), or on both (W = W_sigma x W_qual). We chose the latter case. The user provides the number, Ns, of functions supposed to be needed to model the data (i.e., number of earthquakes), and the ranges for earthquake slip, along-fault position of largest earthquake slip, and rupture length. The inner routine then generates Ns triangular functions whose base lengths and apexes are randomly distributed within the ranges above. The lower function represents the most recent earthquake, the second function the penultimate event, and so on (Supplementary Fig. 13). For each offset data, the routine determines which function is closest (Supplementary Fig. 13). It eventually calculates the weighted root mean square (WRMS) and the Akaike criteria (AICC) for the Ns functions.

The intermediate routine repeats the inner routine Nt times. Nt can be any number (yet calculation time increases with Nt). However, 100 is a fair compromise. At the end of the Nt calculations, the intermediate routine retains the series of Ns functions that has the lowest WRMS.

The outermost routine repeats the intermediate routine Nr times, which allows verification that the calculations are random and the “best” series of functions derived from the intermediate routine are independent from each other. Nr can be any number (yet calculation time increases with Nr), but 100 is a fair compromise. At the end of the outermost routine, Nr “best” functions are defined per earthquake. The routine then calculates the average best function across the Nr best realizations for each of the Ns triangles. These Ns best averaged functions are assigned the closest offset data. Uncertainties on their parameters (rupture length, amplitude and zone of largest earthquake slip) are the standard deviation of the Nr best realizations. The routine eventually calculates the slip difference between two successive averaged best functions, which provides the envelope shape of the earthquake slip profiles.

We made a series of tests to evaluate the impacts of the input parameters (Supplementary Figs. 14–16). Whatever the input range, the position of the largest earthquake slip along the fault is remarkably stable in a 30–45 km window. Eight functions and a rupture length of up to ~110 km are needed to best fit the data while recovering a realistic 1855 earthquake slip. A large 0–22 m range can be adopted for earthquake slip search. This large slip range ensures that the code searches for the smallest to the largest possible earthquakes.

## Supplementary information

Supplementary information.

Data spreadsheet.

## Data Availability

The offset data generated and analyzed during the current study are tabulated and provided in Supplementary Spreadsheet. The code Paleo_Slip-Length is available at https://github.com/ClementPerrin/Paleo_Slip-Length.git. The Lidar data will be placed in early 2020 onto the OpenTopography repository (https://opentopography.org/). All intermediate data processing and calculations generated and analyzed during the current study are available from the corresponding author on reasonable request.
