# Supplementary material for: Repeated giant earthquakes on the Wairarapa fault, New Zealand, revealed by Lidar-based paleoseismology
Source: Sci Rep. 2020 Feb 7;10:2124. doi: 10.1038/s41598-020-59229-3 (PMC7005692; doi:10.1038/s41598-020-59229-3)
Supplement: Supplementary file 1 — Supplementary information. [file 41598_2020_59229_MOESM1_ESM.pdf]

Repeated giant earthquakes on the Wairarapa fault, New Zealand, revealed by Lidar-based paleoseismology

Manighetti I.[1]*, Perrin C.[2], Gaudemer Y.[2], Dominguez S.[3], Stewart N.[1], Malavieille J.[3], and Garambois S.[4]

* Corresponding author I. Manighetti: manighetti@geoazur.unice.fr

SUPPLEMENTARY INFORMATION

Captions of Supplementary figures, data spreadsheet and document A

Supplementary Figure 1: Lidar data, fault mapping and distribution of the ~650 identified offset markers

The airborne Lidar data are shown under different representations. The TLS Lidar data (3 sites) are presented in top left quadrant of the figure. Our mapping of the fault trace is shown in black (no hierarchy in the traces however, due to format transfer issues), with all identified fault segments indicated (AB, CD, etc.) with arrows pointing to their boundaries; they basically form left-stepping echelons. The ~650 identified offset markers are shown in the colors reported in Supplementary Spreadsheet, so as to help the reader to discriminate them. Some of these offset markers are shown in the close-up images in top left and bottom right quadrants of the figure. They are also located with yellow stars and their name (as in Supplementary Spreadsheet) along the fault. Note that, on the fault mapping, barbs indicate vertical slip, not necessarily normal. All the figures have been done with GMT (Wessel et al., Generic Mapping Tools: Improved Version Released, Eos Trans. AGU, 94 (45), 2013).

Supplementary Figure 2: Examples of automatic 3D-Fault-Offset measures

The 5 marker examples are located in Supplementary Figure 1, and shown reconstructed in horizontal plane in Supplementary Figure 3. Their complete measures are in Supplementary Spreadsheet.

Here are shown major steps of automatic offset measurements:

(a): polygon manual drawing (yellow) to roughly locate the 2 offset marker sections across the fault (blue). The code automatically identifies the lower elevation points, which mark the riverbed (in white).

(b): the code then numerically identifies in each polygon, the points of maximum and minimum elevation, and of maximum convexity, slope and concavity. Nine point clouds are

thus identified in each polygon, through which 3D best-fit lines are calculated and projected onto the dipping fault plane. Lateral and vertical offsets are calculated (with their total uncertainties) from the relative position of the best-fit line piercing points on either side of the fault (details in Methods, and in Stewart et al., 2018). The corresponding measurements are listed in Supplementary Table 1, and those retained in analysis indicated below the figure (the others are ignored for they are not well constrained or not geomorphically relevant).
(c): example of 3D view of identified points characterizing one of the marker edges. Green for Max, red for top, blue for Mid, and yellow for base of riser scarp.
(d): horizontal reconstruction of the marker, with the lateral offset derived from the measurements, indicated in figure title.
(e): examples of vertical slip measures and reconstructions across different geomorphic features of the marker. Sign + is where the northern fault compartment has moved up.
All the figures have been done with GMT (Wessel et al., Generic Mapping Tools: Improved Version Released, Eos Trans. AGU, 94 (45), 2013), and MATLAB (Matlab 2019, version 9.7.0 (R2019b). Natick, Massachusetts: the MathWorks Inc.).

Supplementary Figure 3: Examples of marker horizontal backslip reconstructions
The 9 marker examples are located in Supplementary Figure 1, while their complete measurements are in Supplementary Table 1. The examples have been chosen along a central transect across the fault, so as to show the discrete increasing slips (see top figure, copy of Fig.3). Note that, in most examples, the lateral backslip of the targeted marker (green arrow) reconstructs also the pre-faulted geometry of other neighboring markers.
All the figures have been done with GMT (Wessel et al., Generic Mapping Tools: Improved Version Released, Eos Trans. AGU, 94 (45), 2013), and MATLAB (Matlab 2019, version 9.7.0 (R2019b). Natick, Massachusetts: the MathWorks Inc.).

Supplementary Figure 4: Comparison of our measures with prior offset data
Figure 4A: The graphs report all the published and precisely located field offset data we found in the literature (see references in Supplementary Table 1 and in text). Overall, ~70% of our PREF and OPT lateral and vertical offsets are consistent with the prior field measures.
Figure 4B: In a non-published GNS report, Villamor et al. (2008) compiled about 100 additional lateral offsets measured on the field (plot (a) which is Fig.4A from Villamor et al. (2008)). Yet the offset markers are not located precisely and the uncertainties on the offset measures are not provided. To gain insight on these prior field measures, we digitalized the graph (a). The recovered approximate lateral offset values and their along-fault locations are reported on the graph (b) (along with the best constrained field measures from our Fig. 4A). For comparison, the lateral offsets measured in the present study are shown alone in plot (c). Plot (d) shows all prior and present lateral offsets together. Plot (e) allows a better comparison between prior and present lateral offset data: it shows that most prior lateral field offsets fall within one or other of the lateral offset clusters we identified and modeled in the present study (model from Fig.3b).

Supplementary Figure 5: Statistics of the offset measures
(a-top) Distribution of the PREF and OPT lateral offsets across the main and secondary faults. Note that the few lateral offsets greater than 260 m are not presented for clarity.
(a-bottom) Distribution of the PREF and MID vertical offsets across the main and secondary faults. Note that the few vertical offsets greater than 20 m are not presented for clarity. OPT vertical offsets are not shown for they generally are not representative of the actual vertical offset: the vertical offset indeed greatly varies among the geometric features of a marker (the riverbed is especially eroded with a lower apparent vertical offset), and therefore the OPT

mean value is not meaningful. The MID geometric feature has been shown to best record the actual vertical offset. See more detailed explanations in Stewart et al., 2018.
(b-top): Uncertainties on the PREF and OPT lateral offsets.
(b-bottom): Uncertainties on the PREF and MID vertical offsets.

Supplementary Figure 6: Complete PREF lateral offset data over range 0-250 m across the main fault (MF)
The three qualities are shown with different symbols (data in Supplementary Table 1). The orange symbols at top of the graphs indicate fault strike at each offset site. Note the pronounced fault strike change where the lateral offsets are largest.

Supplementary Figure 7: Complete OPT lateral offset data over range 0-250 m across the main fault (MF)
The three qualities are shown with different symbols (data in Supplementary Table 1). The orange symbols at top of the graphs indicate fault strike at each offset site (from Supplementary Table 1). Note the pronounced fault strike change where the lateral offsets are largest.

Supplementary Figure 8: Generic triangular envelope shape of earthquake slip-length profiles
(a) Systematic, asymmetric, triangular slip-length function observed for 44 historical continental earthquakes of Mw ≥ ~6 (left plot, surface data, modified from Manighetti et al., 2005), ~70 historical continental and subduction earthquakes of Mw ≥ ~6 (central plot, depth data derived from source models, modified from Manighetti et al., 2005), and ~30 historical continental earthquakes of Mw ≥ ~6 (right plot, surface data, modified from Perrin et al., 2016). Average generic envelope shape is in yellow.
(b) Variable degree of asymmetry revealed in the earthquake slip profile populations shown in (a); while most slip profiles are asymmetric, some are fairly symmetric, yet still triangular overall. Average generic envelope shape is in yellow.
(c) Additional slip-length profiles of continental and subduction earthquakes, that were not included in plots in (a) and (b) above. Some profiles are asymmetric, others are more symmetric.

Supplementary Figure 9: Modeling of the 3 quality-PREF lateral offsets across the main fault (MF)
(a) PREF lateral offset data (from Supplementary Table 1)
(b) Modeling with parameters indicated at top of the figure (see text and Methods). Dmax C is largest cumulative slip measured per function. The model parameters and uncertainties are reported in Supplementary Table 2.
(c) Earthquake slip profiles derived from the modeling in (b). The model parameters and uncertainties are reported in Supplementary Table 2.

Supplementary Figure 10: Modeling of the 2 and 3 quality-OPT lateral offsets across the main fault (MF)
(a) OPT lateral offset data (from Supplementary Table 1)
(b) Modeling with parameters indicated at top of the figure (see text and Methods). Dmax C is largest cumulative slip measured per function. The model parameters and uncertainties are reported in Supplementary Table 2.
(c) Earthquake slip profiles derived from the modeling in (b). The model parameters and uncertainties are reported in Supplementary Table 2.

Supplementary Figure 11: Modeling of the 2 and 3 quality-PREF lateral offsets across the main fault (MF) and the Secondary faults (SF)
(a) PREF MF and SF lateral offset data (from Supplementary Table 1)
(b) Modeling with parameters indicated at top of the figure (see text and Methods). Dmax C is largest cumulative slip measured per function. The model parameters and uncertainties are reported in Supplementary Table 2.
(c) Earthquake slip profiles derived from the modeling in (b). The model parameters and uncertainties are reported in Supplementary Table 2.

Supplementary Figure 12: Modeling of the 2 and 3 quality-OPT lateral offsets across the main fault (MF) and the Secondary faults (SF)
(a) OPT MF and SF lateral offset data (from Supplementary Table 1)
(b) Modeling with parameters indicated at top of the figure (see text and Methods). Dmax C is largest cumulative slip measured per function. The model parameters and uncertainties are reported in Supplementary Table 2.
(c) Earthquake slip profiles derived from the modeling in (b). The model parameters and uncertainties are reported in Supplementary Table 2.

Supplementary Figure 13: Basic elements of the offset data modeling code
(a) Search for the best fitting triangular functions (see Methods)
(b) Data assignment to closest best-fitting function

Supplementary Figures 14 to 16: Test of impact of input parameters in offset data modeling code
Fig.14A: Influence of along-fault distance range Ls for searching the apex position of each triangular function. (b) Models run with different Ls range, indicated in bold on top of the graphs. Apart from Ls, models are run with the conditions reported on top of the graphs (a) Statistics of the models. The range Ls (example [0-30 km]) is represented in X axis with its half value (for the example above: 15 km). On the 2 top graphs, red is mean value of WRMS or AICC. In third graph, red is 1855 earthquake slip.
The test shows that, whatever the range of Ls, the position of largest slip is found stable (provided the Ls range contains it), between 30 and 45 km, and more precisely at about 37-39 km. The best model in terms of indicators is for the Ls range [30-45 km].

Fig.14B: Influence of number of functions input to fit the data. (b) Models run with different numbers of functions, indicated in bold in the graphs. Apart from the number of functions, models are run with the conditions reported on top of the graphs (a) Statistics of the models. On the 2 top graphs, red is mean value of WRMS or AICC. In third graph, red is 1855 earthquake slip.
The test shows that, as expected, the statistical indicators of the models (WRMS and AICC) decrease with the number of functions. Yet, only the range [3-8] functions recovers a realistic 1855 earthquake slip. In this range, the model with 8 functions is the most robust statistically.

Fig.15: Influence of the slip increment range to search for the slip of each function. (b) Models run with different slip increment amplitudes, indicated in bold in the graphs. Apart from the slip increment amplitude, models are run with the conditions reported on top of the graphs (a) Statistics of the models. On the 2 top graphs, red is mean value of WRMS or AICC. In third graph, red is 1855 earthquake slip.

It first needs to be noted that no continental earthquake is known to have produced more than 18-19 m of slip at surface (see Rodgers and Little, 2006; Manighetti et al., 2007). Therefore slip increments provided as inputs to the model cannot be much greater than 20 m.
The test shows that realistic 1855 and prior earthquake slips are only produced with slip increment ranges from [0-18m] to [0-24m], while the model indicators are better for the larger ranges. The best compromise is found for the slip increment range [0-22 m], as the later does not produce any earthquake slip greater than 20 m, as does the range [0-24 m].

Fig.16: Influence of the rupture length range either side of the Lidar stretch. (b) Models run with different rupture length ranges either side (total rupture length indicated in bold in the graphs). Apart from the length range, models are run with the conditions reported on top of the graphs (a) Statistics of the models. On the 2 top graphs, red is mean value of WRMS or AICC. In third graph, red is 1855 earthquake slip.
The test shows that, the longer the rupture, the better the indicators of the models. However, this is mainly related from the longer rupture-models integrating more data points. It is therefore difficult to determine the actual rupture length. The model statistics shows however that the modeled 1855 earthquake slip abruptly drops down for rupture length greater than 110 km. This might suggest it is unlikely that the total rupture length be greater than 110 km.

Supplementary Figure 17: Lateral and vertical slip profiles of the 8 past large earthquakes on the Wairarapa fault, derived from modeling of PREF offset data
Data from Supplementary Table 1
(a) Lateral slip profiles. In all graphs, the profiles show the lateral offset data which have been assigned to one or other of the 8 modeled functions (from Figs.3b and Supplementary 10; only quality 1 and 2). Therefore, the first, lower profile in red is the 1855 earthquake lateral slip profile, while the above profiles are cumulative. The corresponding earthquakes are indicated. The vertical lines highlight the major segments (from Supplementary Fig. 1) which seem to shape the lateral slip distributions, especially the 1855 one. The maximum and mean slips of the 1855 earthquake are indicated (derived from the slip data in red).
(b) Vertical slip profiles. The lateral offset data characterizing each earthquake being identified in (a), we could isolate the corresponding vertical slip data. Those are plotted in (b). Therefore, the first, lower profile in red is the 1855 earthquake vertical slip profile, while the above profiles are cumulative. The corresponding earthquakes are those indicated in (a). The vertical lines are as in (a). The mean vertical slip of the 1855 earthquake is indicated (derived from the slip data in red), while the mean cumulative vertical slips of the prior earthquakes are also indicated (derived from the data shown).

Supplementary Figure 18: Vertical slips measured along the fault length for the entire dataset
PREF vertical offset data from Supplementary Table 1, on both Main (in black) and Secondary faults (in grey).
Distance along the fault is measured from west Lidar stretch. Vertical slips are shown with their sense of slip, positive where the northern fault compartment is up, and negative where the northern fault compartment is down.
Fault strikes are shown in orange (MF), and blue (Sec. faults).

Supplementary Figure 19: Relation between lateral and vertical slips for the entire dataset
PREF lateral and vertical offset data from Supplementary Table 1, on main fault (qualities 1 and 2 only).

Overall, the cumulative vertical slips increase with the cumulative lateral slips.

Supplementary Figure 20: Lateral to vertical slip ratios, and composed total slip profiles of the 5 most recent large earthquakes on the Wairarapa fault
Data from Supplementary Table 1
(b) Vertical to lateral slip ratios for the 5 most recent earthquakes. The lateral and vertical slip data are those from Supplementary Fig. 17. Therefore, the first, lower plot in red shows the vertical to lateral slip ratio along the 1855 earthquake rupture, while the above graphs show the cumulative vertical to lateral slip ratios along the prior earthquake ruptures. The corresponding earthquakes are indicated in (b). The vertical lines are as in Supplementary Fig. 17. The mean vertical to lateral slip ratios are indicated.
(b) Total, composed slip profiles of the 5 most recent earthquakes. The lateral and vertical slip data are those from Supplementary Fig. 17. They have been composed to produce the total slip profiles. The first, lower profile in red is the 1855 earthquake total slip profile, while the above profiles are cumulative total profiles. The corresponding earthquakes are indicated. The vertical lines are as in Supplementary Fig. 17. The maximum total slips are indicated.

Supplementary Figure 21: Lateral to vertical slip ratios, and composed total slip profiles of the 8 past large earthquakes on the Wairarapa fault
Same figure as Supplementary Fig. 20 but for the 8 identified earthquakes.

Supplementary data spreadsheet (excel file): the ~15, 800 offset measurements provided in this study

Markers are named (column A) in reference to the coordinate of their northern piercing point in the Lidar data reference. Note however that, during the measurement process, a few changes were made to some of the marker traces while their initial name was not changed. Markers are located on Supplementary Fig.1 with the color indicated in column E. In column A, markers measured in 1 m airborne Lidar data are in white background, while markers measured in 30 cm TLS Lidar data are in orange background. Some markers have both measurements. Prior field offset measures are indicated in blue.

Distance (column B) is measured along major fault trace from its westernmost end in Lidar data. For secondary faults, distance is projected on main fault trace. Maximum error on distances is ~20 m.

Major fault segments (column C) are derived from fault mapping, and reported on Supplementary Fig.1.

Column D discriminate markers offset across major fault trace (orange background color in column G) or secondary faults (blue background color in column G). Names of secondary faults are not reported in Supplementary Fig.1 for clarity.

Marker brief description is provided in Column F, with additional comments in Column H. Fault, especially sense of dip at surface, is commented in Column I.

Quality rating in Column G is based at this stage on preservation of the marker and clarity of its lateral offset. This quality is updated later in Columns BX and BZ.

Columns J to AY are output measurements from the 3D-Fault-Offset code; the P-AG columns are the lateral offset measures, and the AH-AY columns are the vertical offset measures. See Stewart et al. (2018) for more explanations. Note that, because the WP fault dips towards the NW, the convention for the manual tracing of the fault line results in a rotation of the original DEM and output measures. Therefore, any "west" and "east" in the code output results refers to actual East and West, respectively, while the output "north" and "south" are actual south

and north, respectively. The output sign of the vertical offsets thus needs to be multiplied by (-1) to obtain the actual sense of vertical slip (positive where north up).

In the J-AY columns, the grey values are those which are not well constrained or not geomorphically-relevant: they are ignored from the analysis, and only the black values, geomorphically-relevant, are retained. Note however that there are a few cases of complex markers for which discriminating geomorphically-relevant offsets is difficult; for those cases we kept all or most of the code measures so as to have a statistically-constrained offset. The "grey ignored values" are defined from the lateral offsets and then applied as "mirror" to the vertical offsets; this is a simplification as vertical offsets are sometimes better defined than their corresponding lateral offsets. The underlined values are the PREF offsets. Note that, when no PREF value can be recognized, the OPT value is retained as PREF. Symbol >> means that the error on the offset is unrealistic (i.e., the code could not resolve a relevant best-fit line).

The prior offset measures and their references are indicated in dark blue, at the place of the corresponding marker.

Column AZ synthesizes the number of geomorphically-relevant lateral (and vertical) offsets used for the calculation of the overall mean OPT lateral (or vertical) offset reported in Column BK (and BO for vertical).

Columns BA to BP reports the offset values most relevant for analysis: lateral offset of riverbed (or mid if riser) in BA; vertical offset of Mid slope in BC (see explanation in Stewart et al., 2018); PREF lateral offset in BE; PREF vertical offset in BG; mean GEOM lateral offset per series of measures in BI; global OPT mean lateral offset in BK; mean GEOM vertical offset per series of measures in BM; global OPT mean vertical offset in BO. Errors are provided at 1 sigma. The data more used in the present analysis are thus in Columns BE-BF, BG-BH, BK-BL, and BO-BP.

Column BQ reports comments on the code results and backslip reconstructions, especially on vertical slips.

Column BW examines whether the PREF and OPT lateral offsets also reconstruct some adjacent markers. If not, the quality of the lateral offset in Column BX is maintained as that first stated in Column G. If yes, the quality is increased because the reconstruction of several markers attests of the robustness of the lateral offset. Dark blue is for quality 1 (best), medium blue for quality 2 (good), and white for quality 3 (weaker). Column BZ evaluates the quality of the PREF vertical slip as both being well constrained and well reconstructing the prefaulted vertical geometry of the marker. Dark green is for quality 1 (best), medium green for quality 2 (good), and white for quality 3 (weaker).

A synthesis of total number of identified markers, series of measures, code offset measures, and geomorphically-relevant measures retained in analysis, is provided at the end of each segment section. A short table synthesizing all these numbers is provided at the bottom of Table 1. In red are the segments where the largest proportions of offsets were measured on secondary faults off the main fault trace.

Supplementary Document A: Parameters of best lateral offset models

Figures where models are shown are indicated in brackets.

The best models are the average models calculated by the outermost routine from the multiple Nr "best models" derived from the intermediate routine (see Methods). Here we generally chose Nr=500, and therefore each of the best models is derived from 50 000 iterations.

Ll is left (west) distance of rupture western tip with respect to main fault western end in Lidar data, taken as 0 km. Lr is right (east) distance of rupture eastern tip with respect to main fault

eastern end in Lidar data, which is 70 km. Ls is position of largest lateral slip along the [0-70 km] main fault in Lidar data. Ds is largest modeled lateral slip. In all cases, error is standard deviation of the Nr models. Errors on Ds are conservative and overestimated because their calculation is not weighted with the quality of the Nr models (i.e., a weaker model with larger WRMS and AICC parameters has same weight as a more robust model with smaller WRMS and AICC).

"Max EQ slip" is largest earthquake lateral slip derived from Ds values. For reasons explained above, we do not translate the Ds errors on the Max EQ slips.

Mean values of parameters are provided for each model, along with the mean WRMS and AICC criteria of the model.

Best Model PREFQ1Q2_MF is calculated from quality 1 and quality 2 PREF lateral offsets (in range 0-160m) measured across the main fault. Data are weighted by both their uncertainty and quality weight. The input parameters for the best model calculation are M_N_fit_rand_tri13(500,100,L,D,W,8,-20,0,30,45,0,22,70,90), that is : Nr=500, Nt=100, 8 functions are searched, range for western rupture tip (with respect to western end of Lidar stretch) is [-20-0 km], range for along-fault search of largest slip is [30-45 km], range for earthquake slip search is [0-22 m], and range for eastern rupture tip (with respect to eastern end of Lidar stretch) is [70-90 km]. These input parameter ranges are the same for the other models below.

Best Model PREFQ1Q2Q3_MF is calculated from quality 1, quality 2 and quality 3 PREF lateral offsets (in range 0-160m) measured across the main fault. Data are weighted by both their uncertainty and quality weight. The input parameters for the best model calculation are M_N_fit_rand_tri13(500,100,L,D,W,8,-20,0,30,45,0,22,70,90) (as above).

Best Model OPTQ1Q2_MF is calculated from quality 1 and quality 2 OPT lateral offsets (in range 0-160m) measured across the main fault. Data are weighted by both their uncertainty and quality weight. The input parameters for the best model calculation are M_N_fit_rand_tri13(500,100,L,D,W,8,-20,0,30,45,0,22,70,90) (as above).

Best Model OPTQ1Q2Q3_MF is calculated from quality 1, quality 2, and quality 3 OPT lateral offsets (in range 0-160m) measured across the main fault. Data are weighted by both their uncertainty and quality weight. The input parameters for the best model calculation are M_N_fit_rand_tri13(500,100,L,D,W,8,-20,0,30,45,0,22,70,90) (as above).

Best Model PREFQ1Q2_MF+SF is calculated from quality 1 and quality 2 PREF lateral offsets (in range 0-160m) measured across both the main fault and the secondary faults. Data are weighted by both their uncertainty and quality weight. Input parameters for the model calculation are M_N_fit_rand_tri13(500,100,L,D,W,8,-20,0,30,45,0,22,70,90) (as above).

Best Model PREFQ1Q2Q3_MF+SF is calculated from quality 1, quality 2 and quality 3 PREF lateral offsets (in range 0-160m) measured across both the main fault and the secondary faults. Data are weighted by both their uncertainty and quality weight. Input parameters for the model calculation are M_N_fit_rand_tri13(100,100,L,D,W,8,-20,0,30,45,0,22,70,90) (as above but with Nr=100).

Best Model OPTQ1Q2_MF+SF is calculated from quality 1 and quality 2 OPT lateral offsets (in range 0-160m) measured across both the main fault and the secondary faults. Data are weighted by both their uncertainty and quality weight. Input parameters for the model calculation are M_N_fit_rand_tri13(500,100,L,D,W,8,-20,0,30,45,0,22,70,90) (as above).

Best Model OPTQ1Q2Q3_MF+SF is calculated from quality 1, quality 2 and quality 3 OPT lateral offsets (in range 0-160m) measured across both the main fault and the secondary faults. Data are weighted by both their uncertainty and quality weight. Input parameters for

model calculation are M_N_fit_rand_tri13(100,100,L,D,W,8,-20,0,30,45,0,22,70,90) (as above but with Nr=100).

Global means in red are average from all model means, calculated with Gaussian probability density functions.

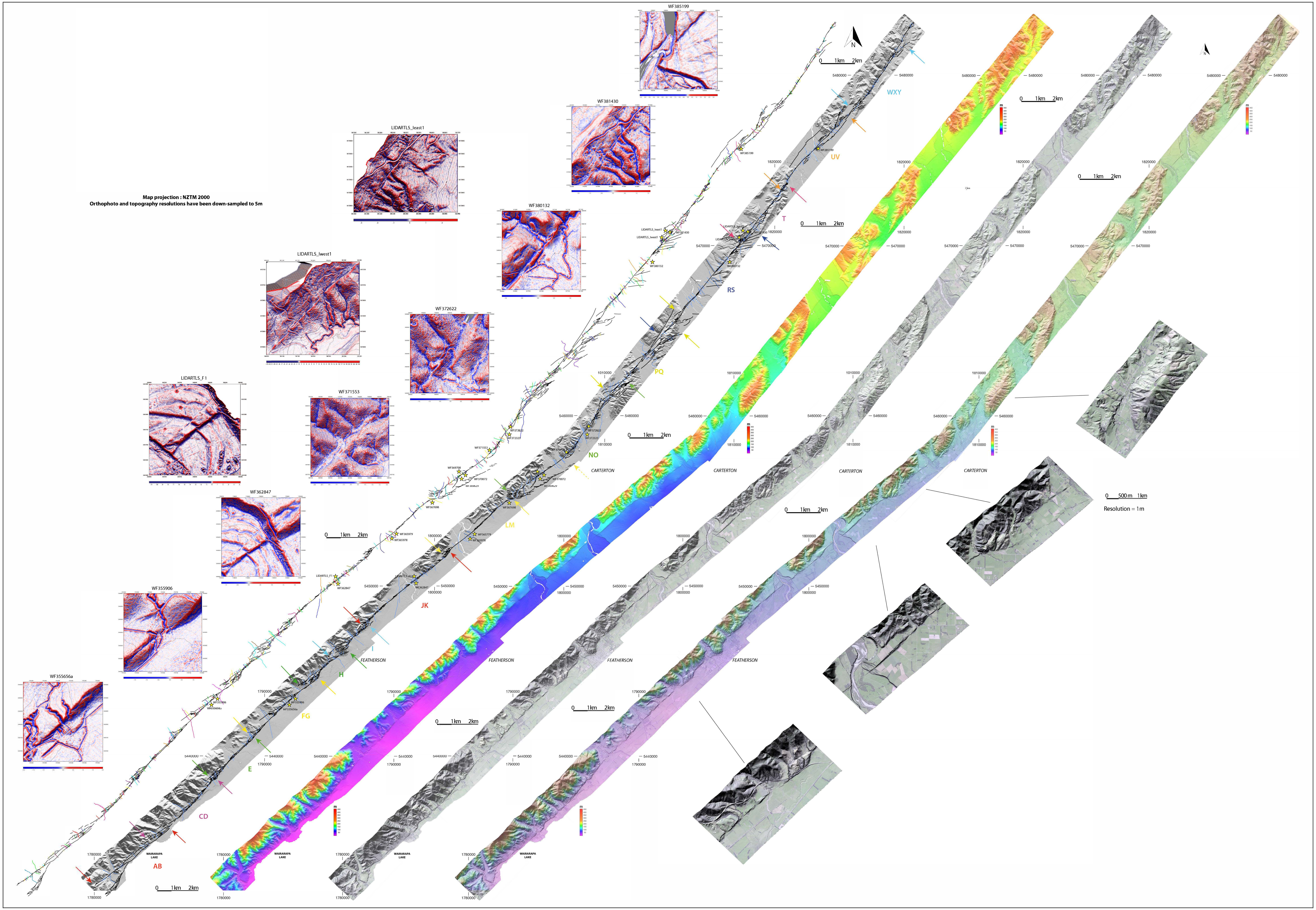

Supplementary Figure 1; Manighetti et al.

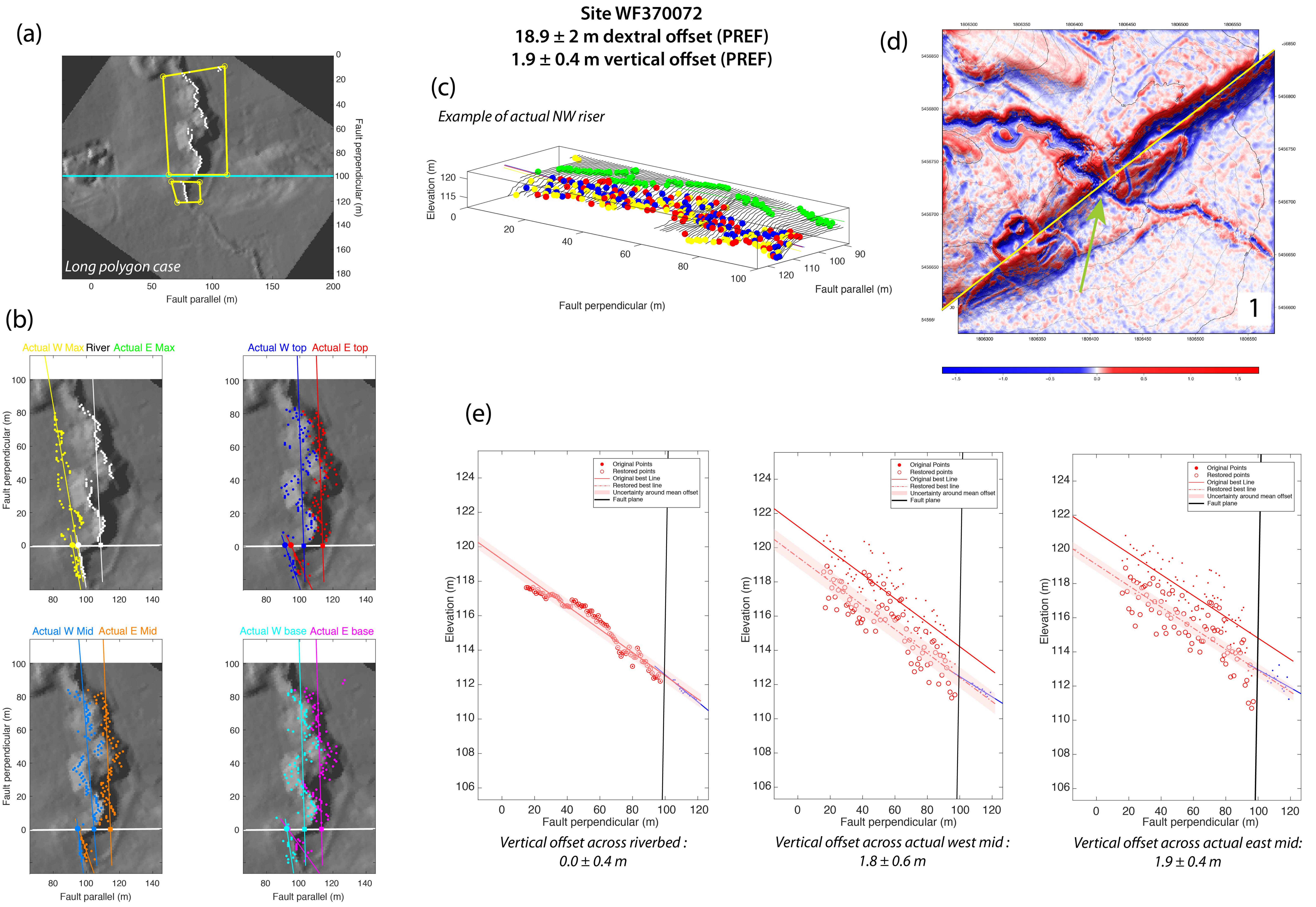

Supplementary Figure 2; Manighetti et al.

Site WF369708
35.0 ± 2.1 m dextral offset (PREF)
-2.8 ± 0.8 m vertical offset (PREF)

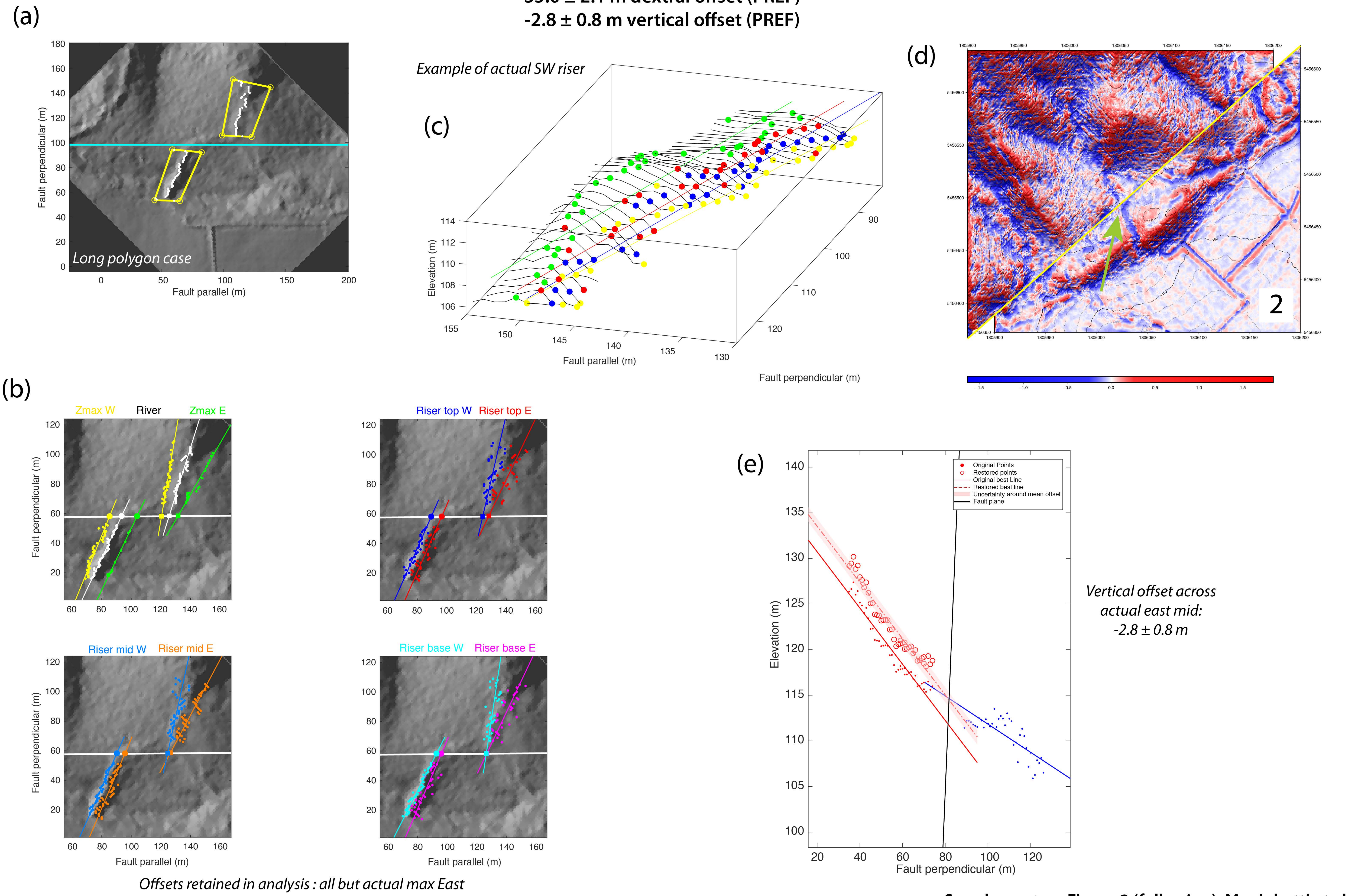

Supplementary Figure 2 (following); Manighetti et al.

Site WF365779

34.8 ± 1.3 m dextral offset (PREF) (prior measure from Carne et al., 2011: 22.1 ± 1.5 m)

9.5 ± 0.5 m vertical offset (PREF) (prior measure from Carne et al., 2011: 2.67 ± 0.05 m)

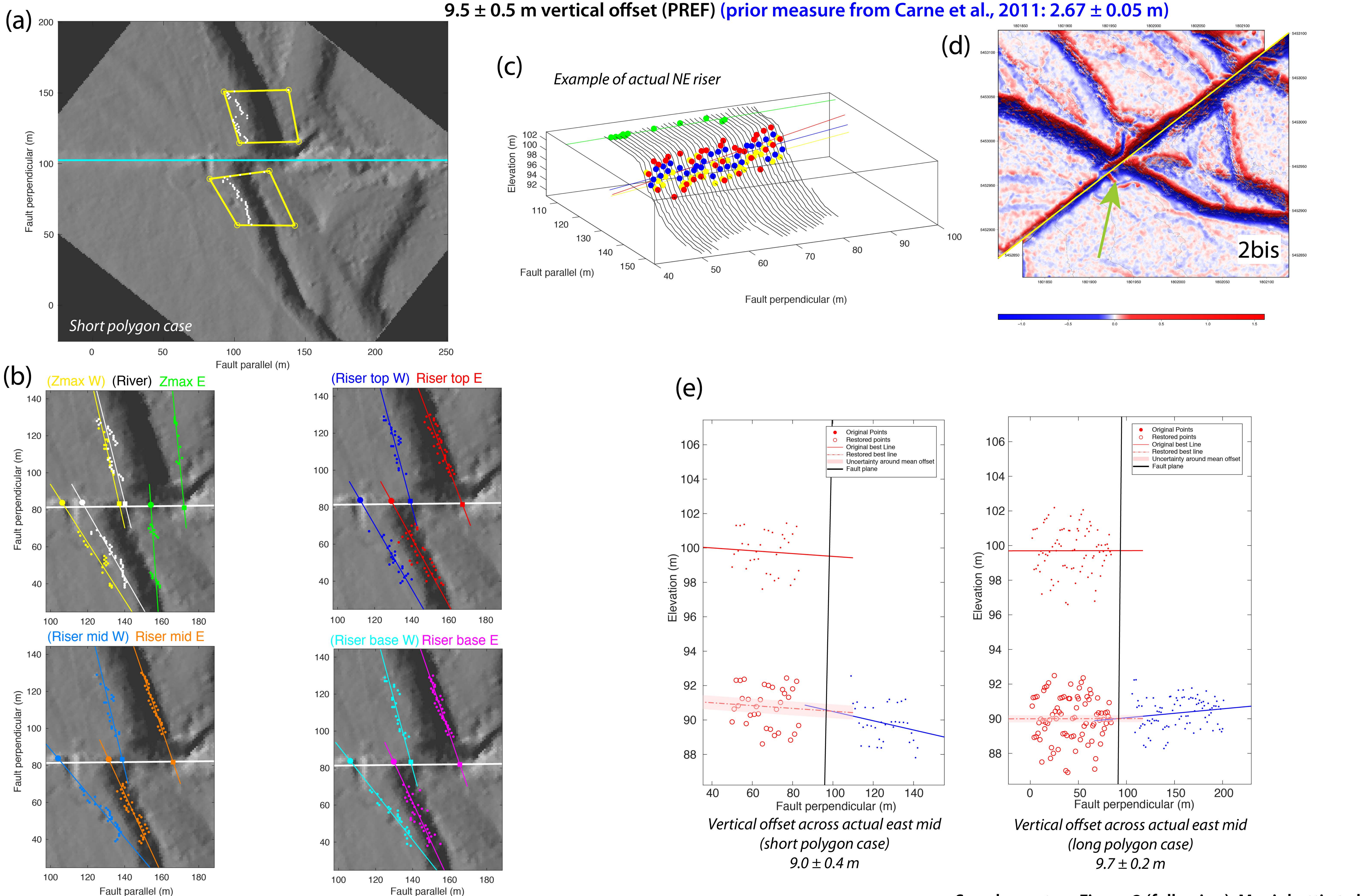

Offsets retained in analysis : actual east bot, mid and top

Supplementary Figure 2 (following); Manighetti et al.

Site WF367698

50.9 ± 2.4 m dextral offset (PREF)

6.9 ± 1 m vertical offset (PREF)

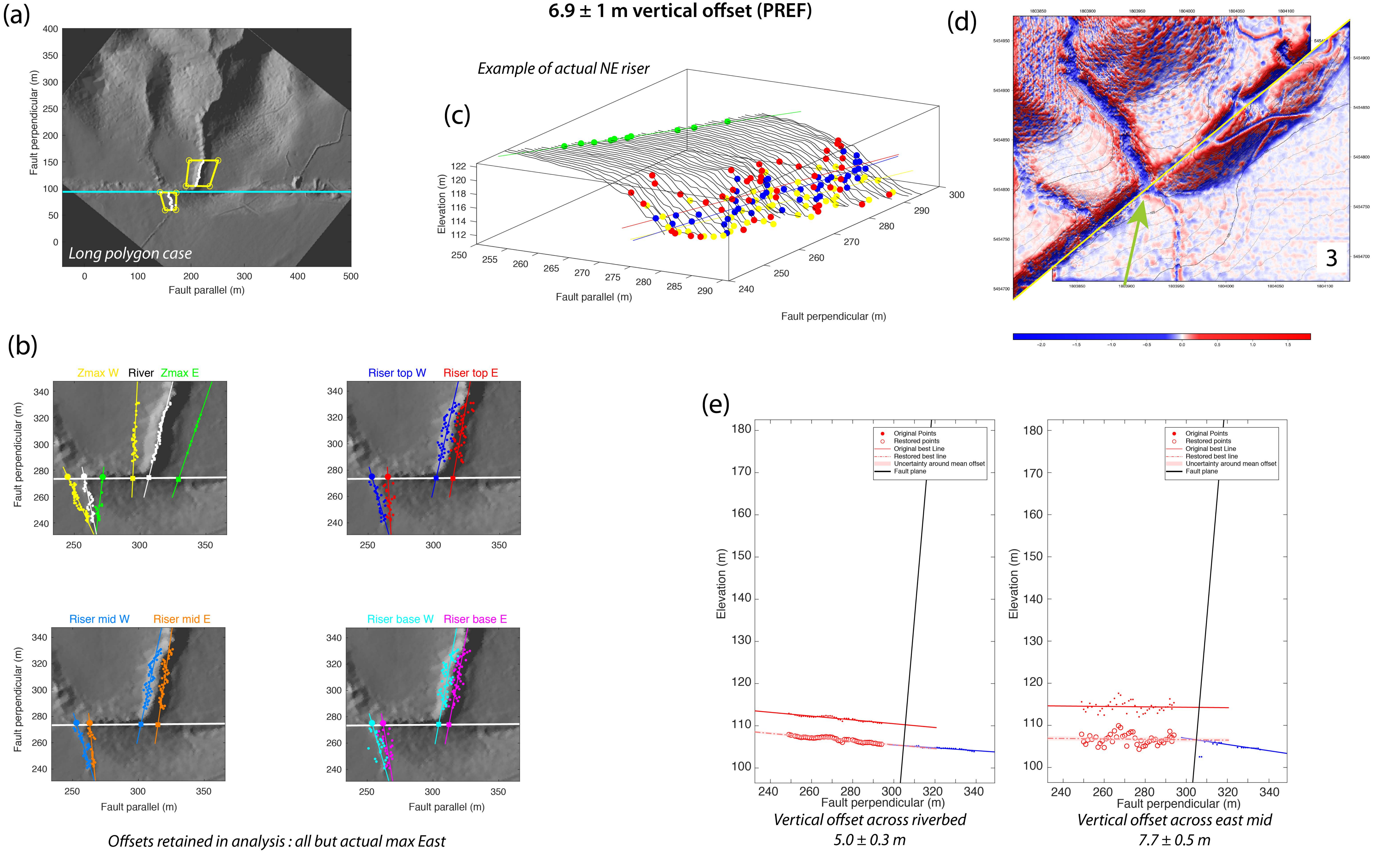

Supplementary Figure 2 (following); Manighetti et al.

Site WF369629
68.8 ± 2.9 m dextral offset (PREF)
-1.7 ± 0.6 m vertical offset (PREF)

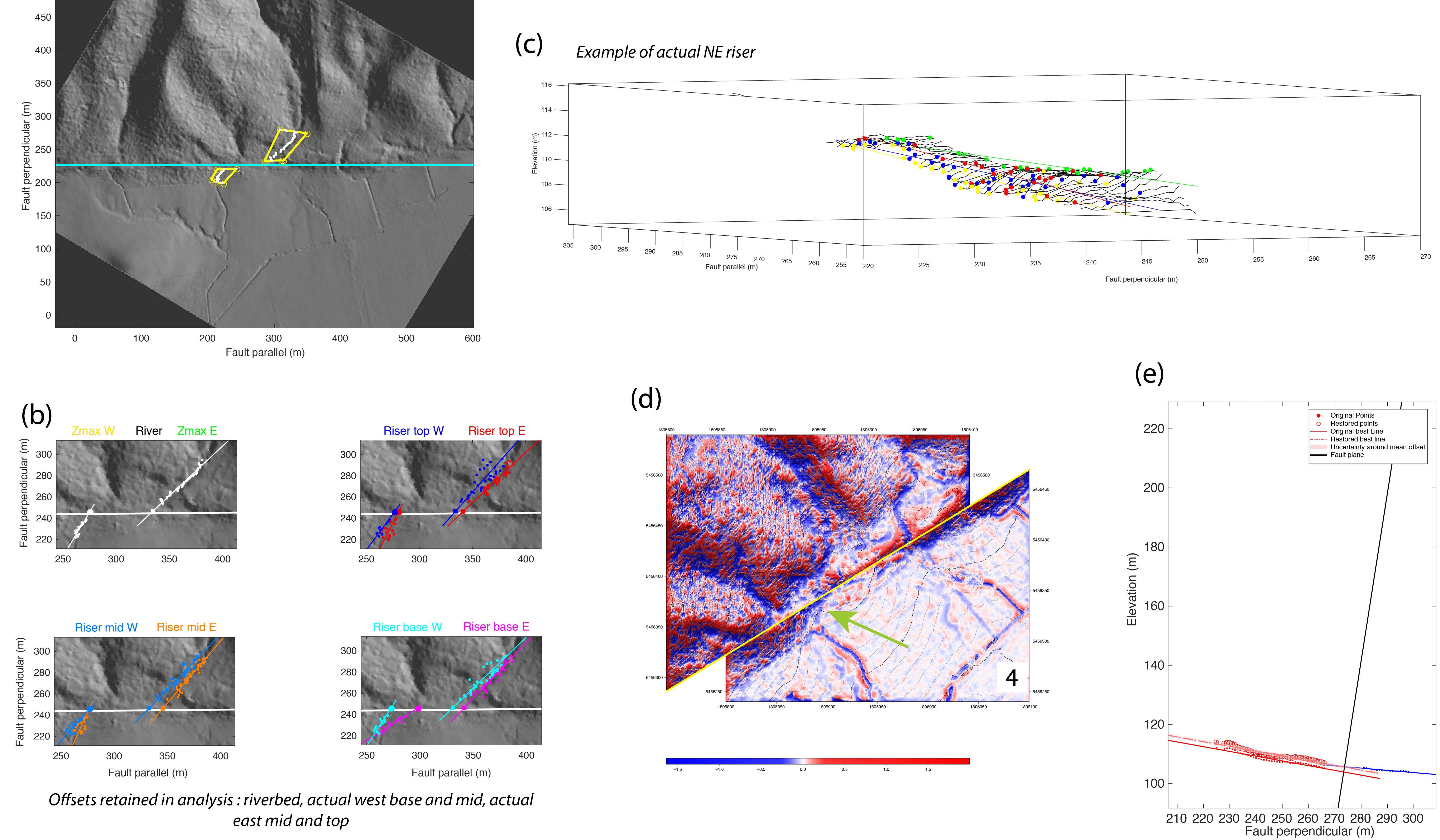

Offsets retained in analysis : riverbed, actual west base and mid, actual east mid and top

Vertical offset across riverbed
-1.7 ± 0.6 m

Supplementary Figure 2 (following); Manighetti et al.

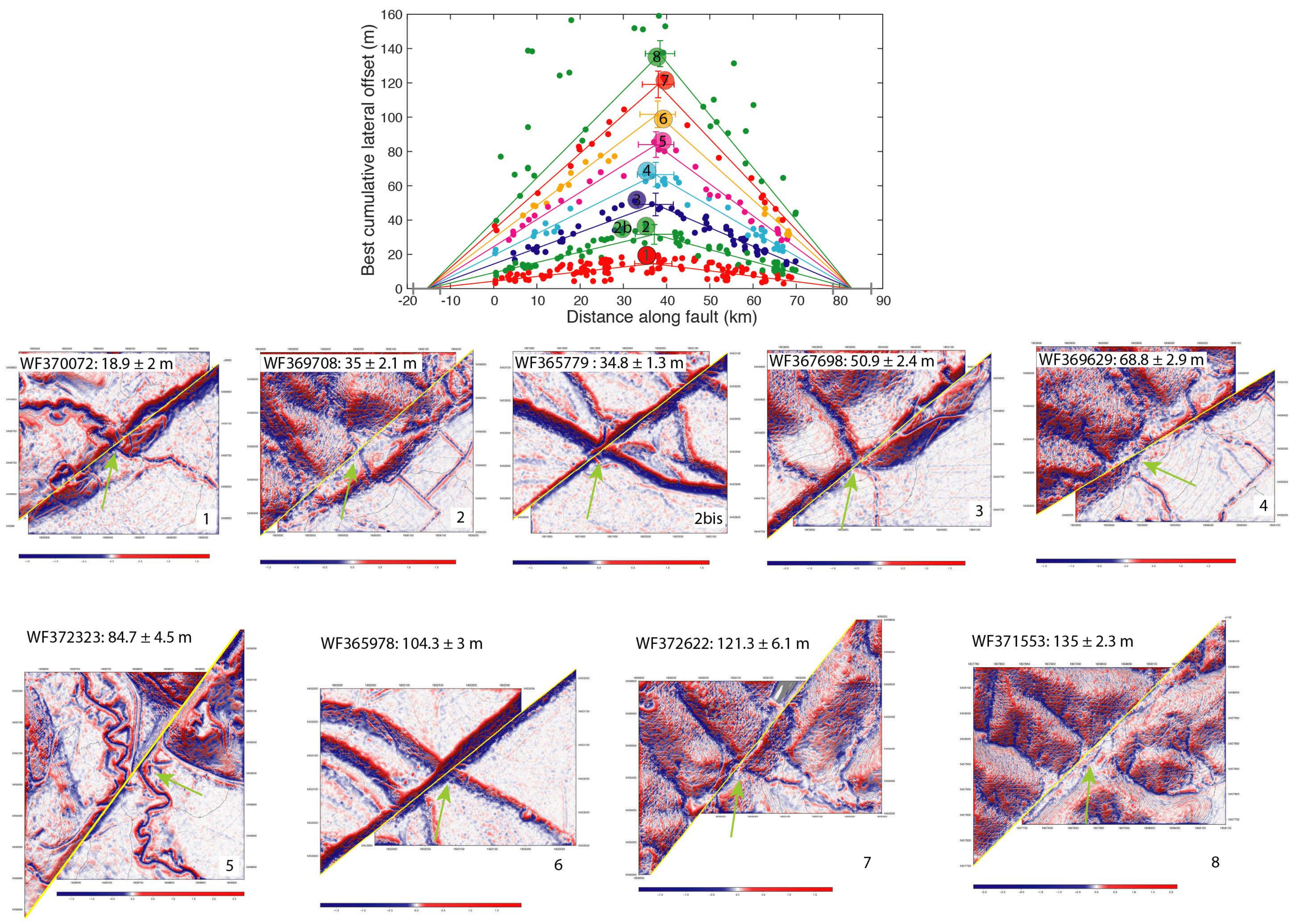

Supplementary Figure 3; Manighetti et al.

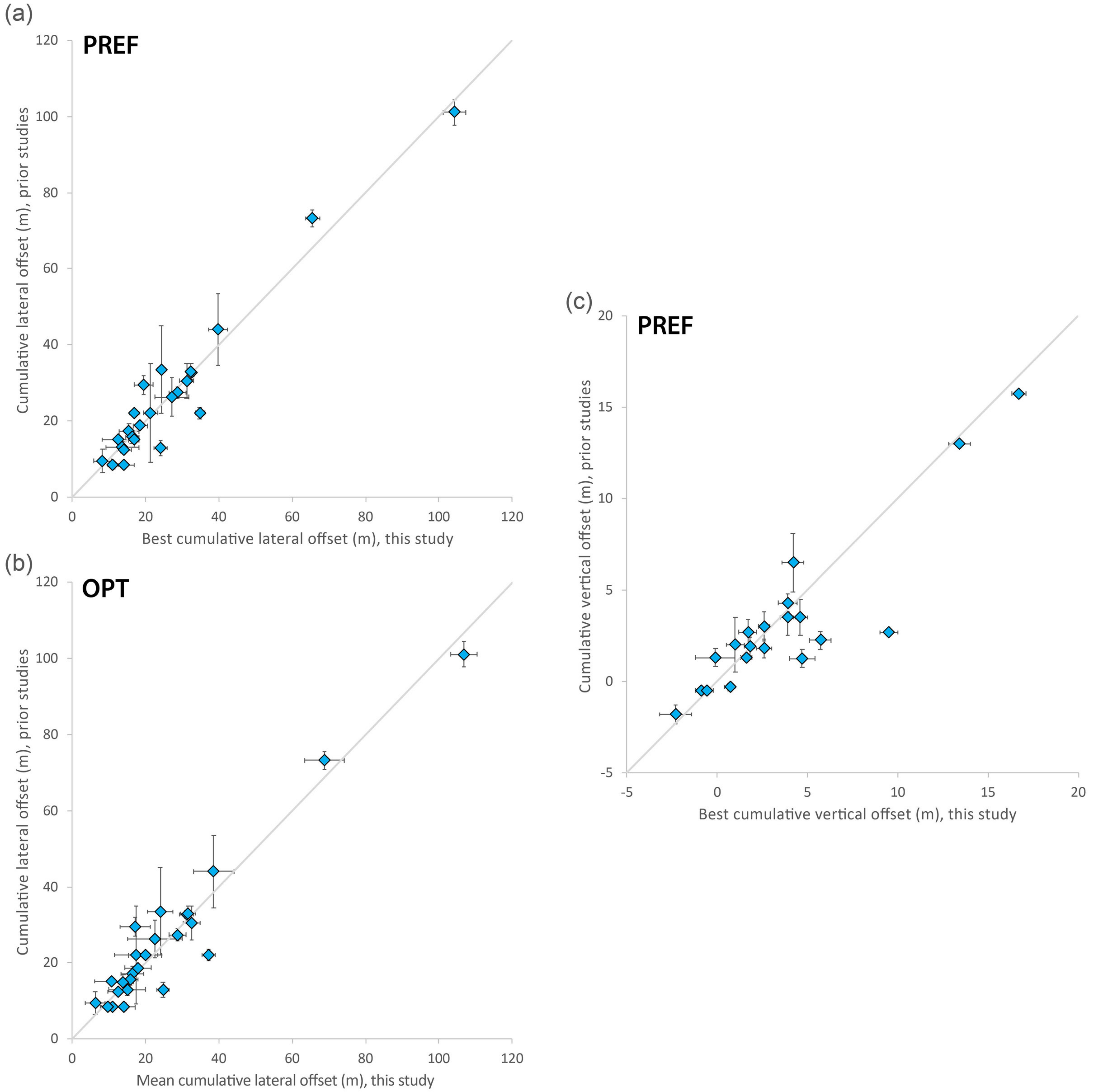

Supplementary Figure 4A- Manighetti et al.

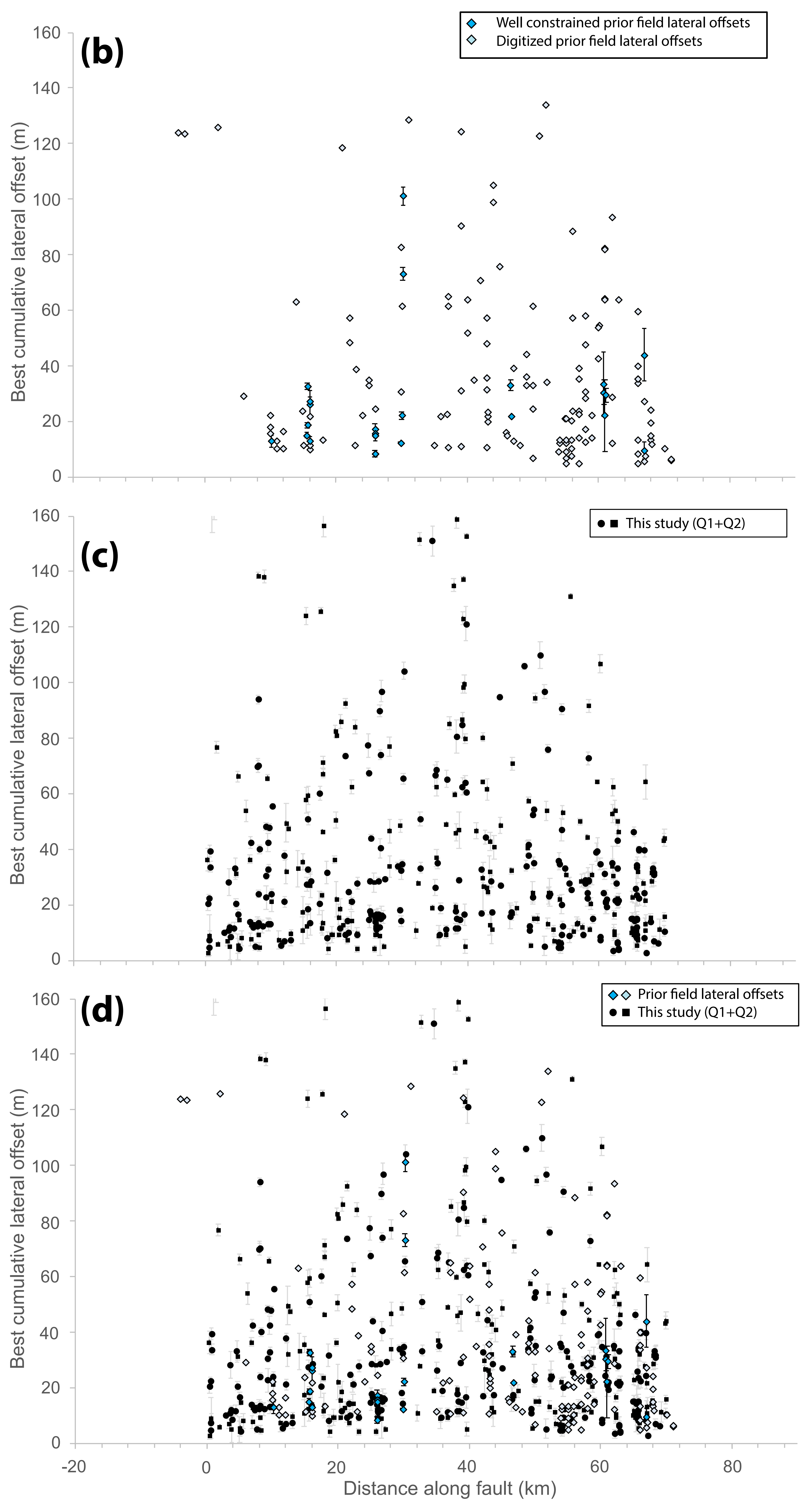

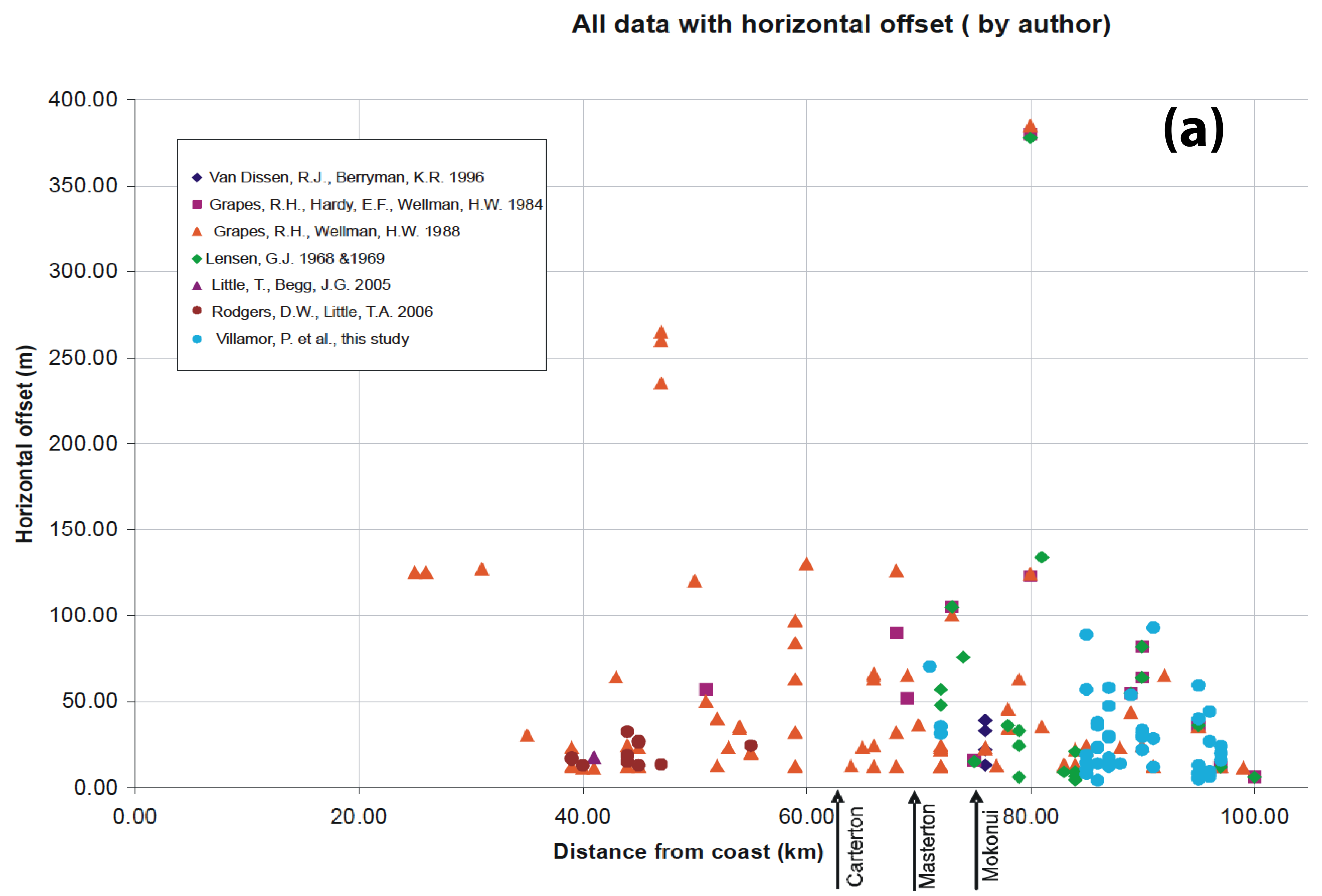

Supplementary Figure 4B- Manighetti et al.

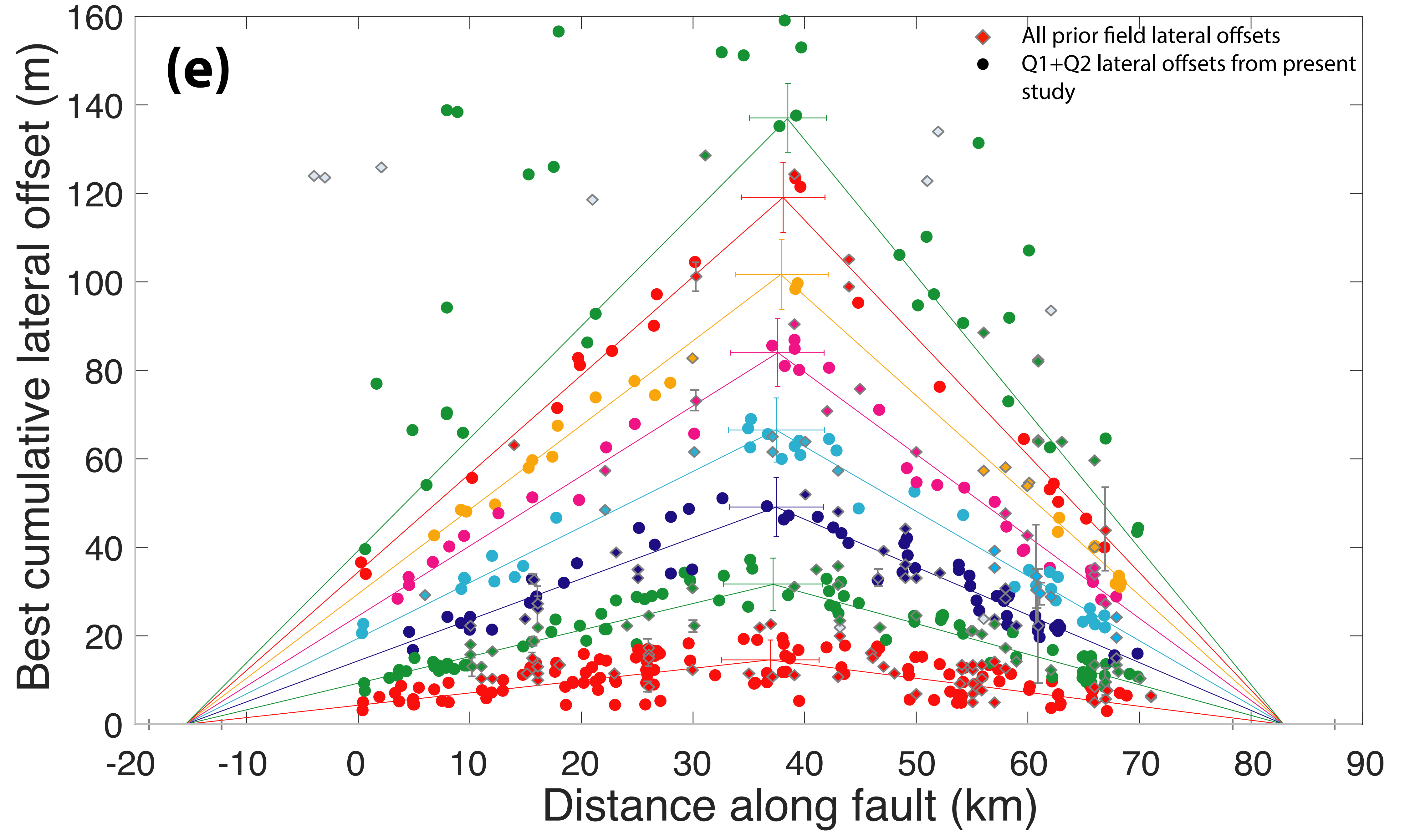

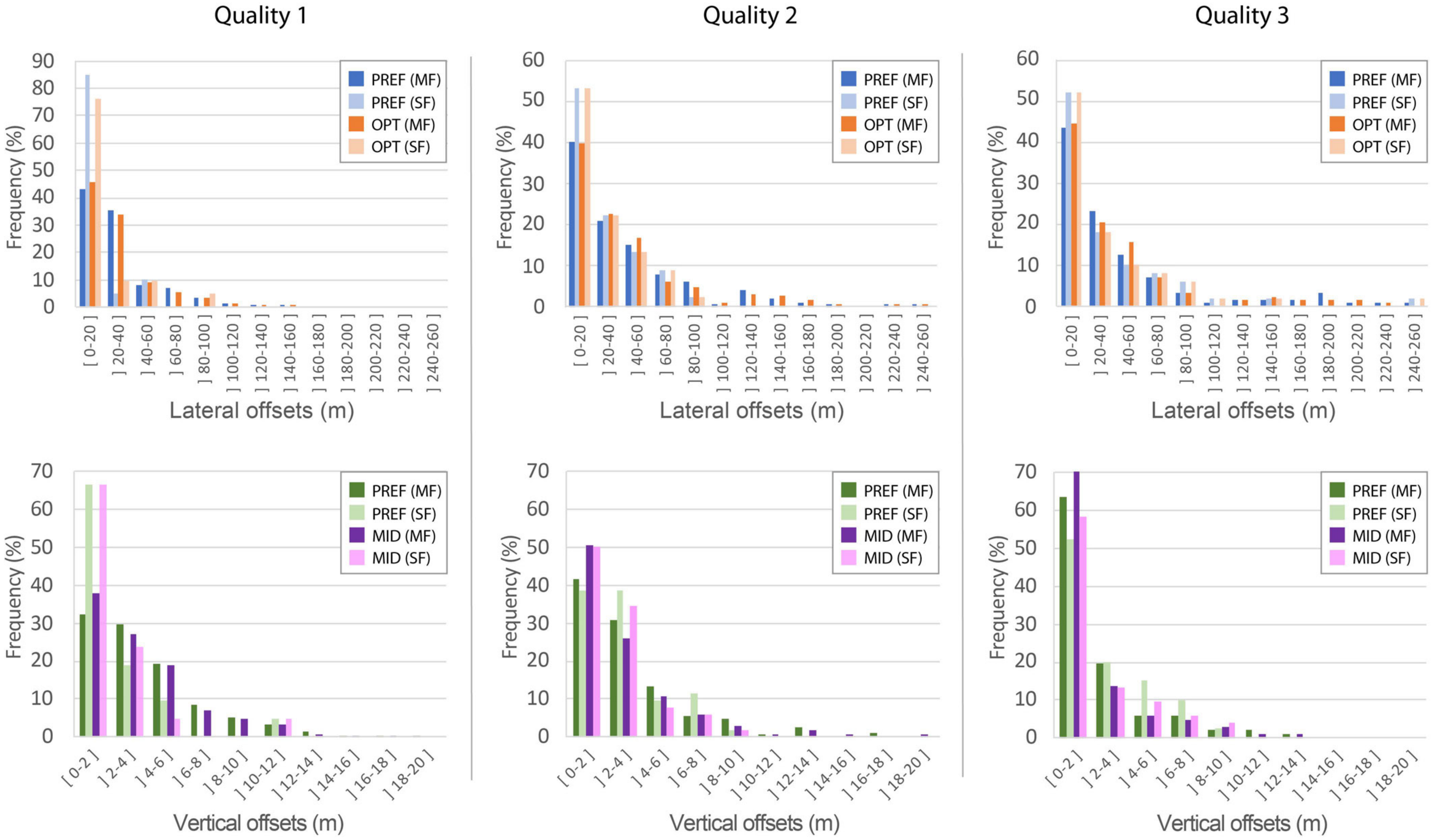

Supplementary Figure 5a; Manighetti et al.

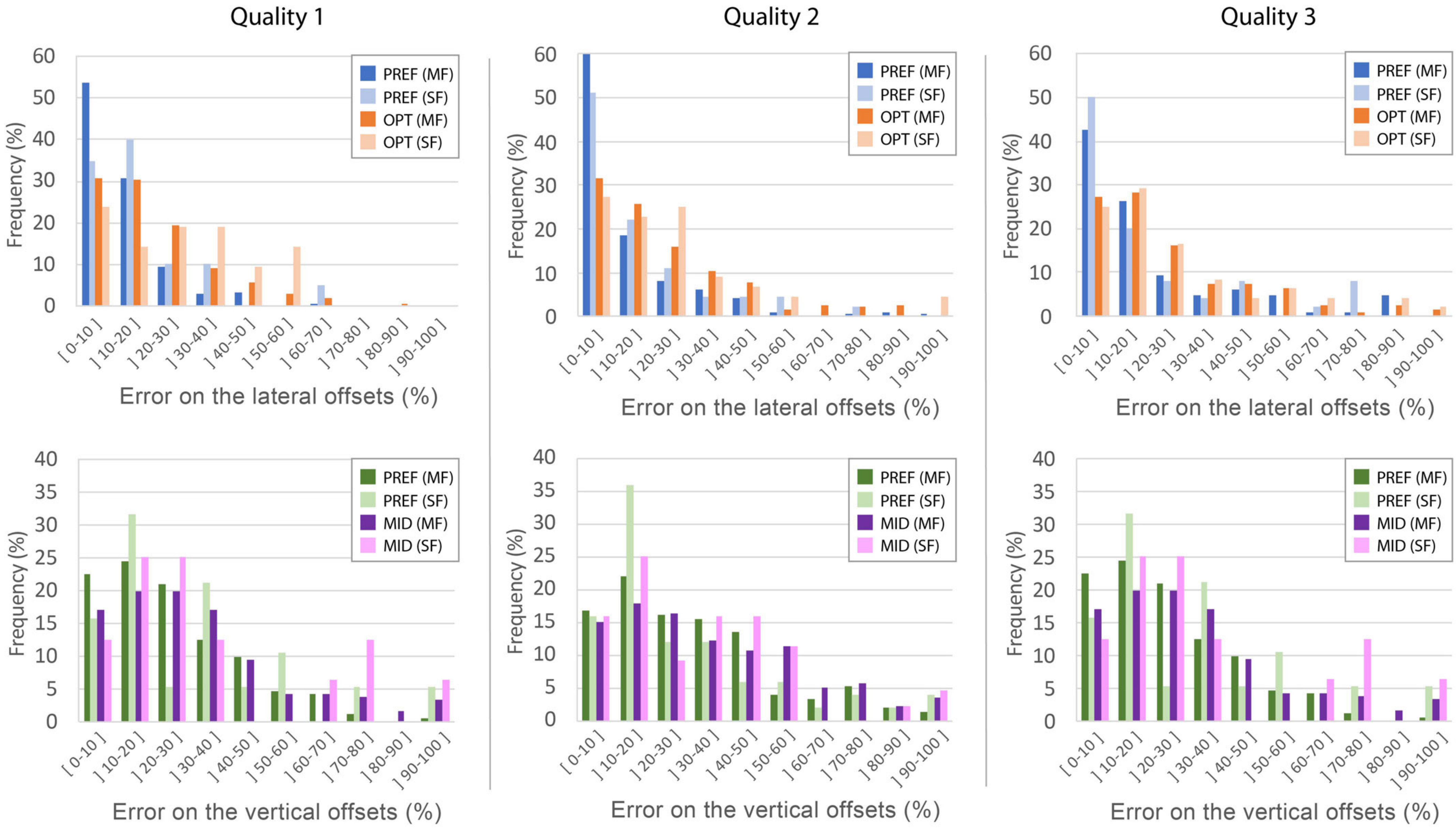

Supplementary Figure 5b; Manighetti et al.

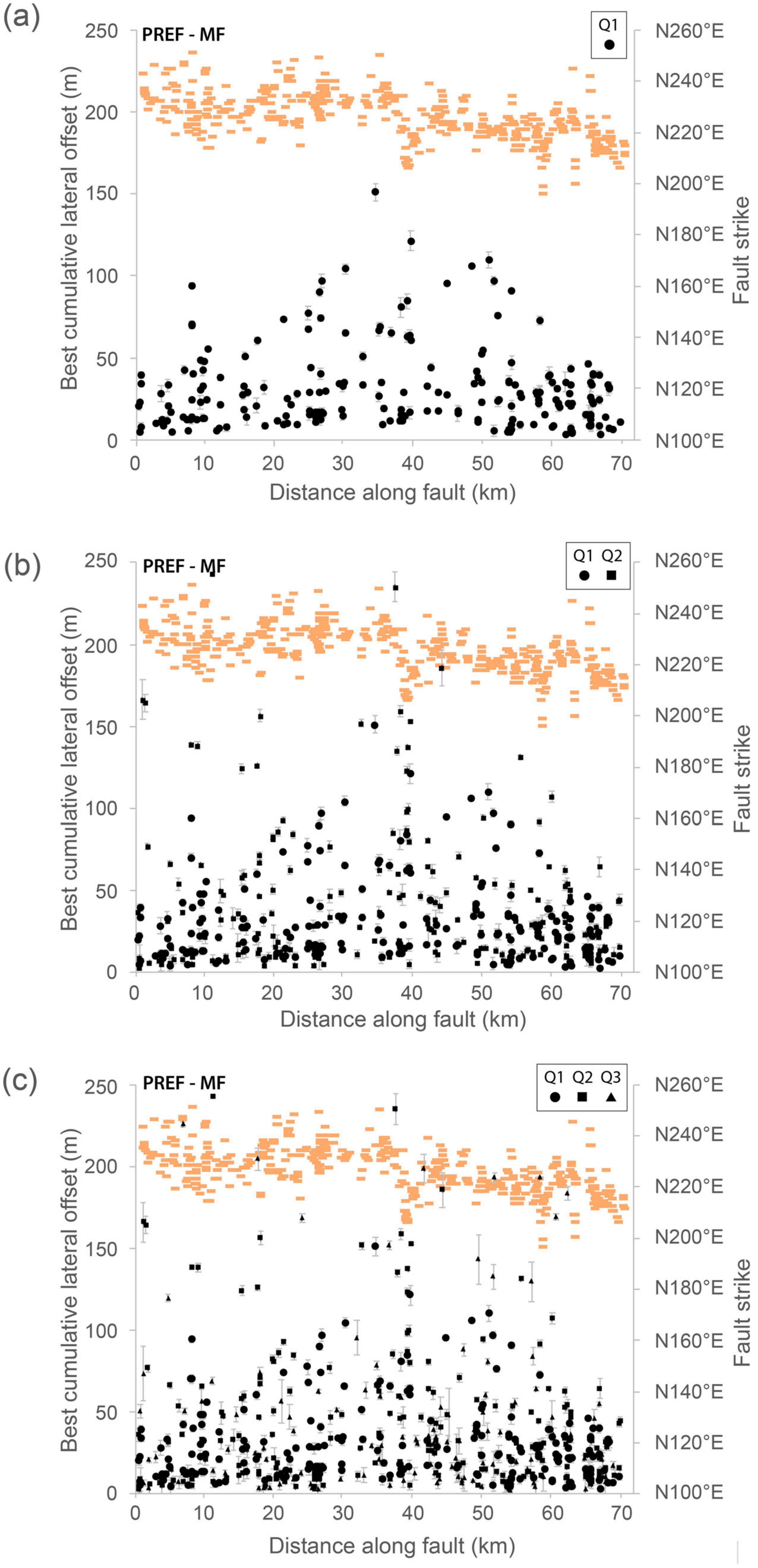

Supplementary Figure 6; Manighetti et al

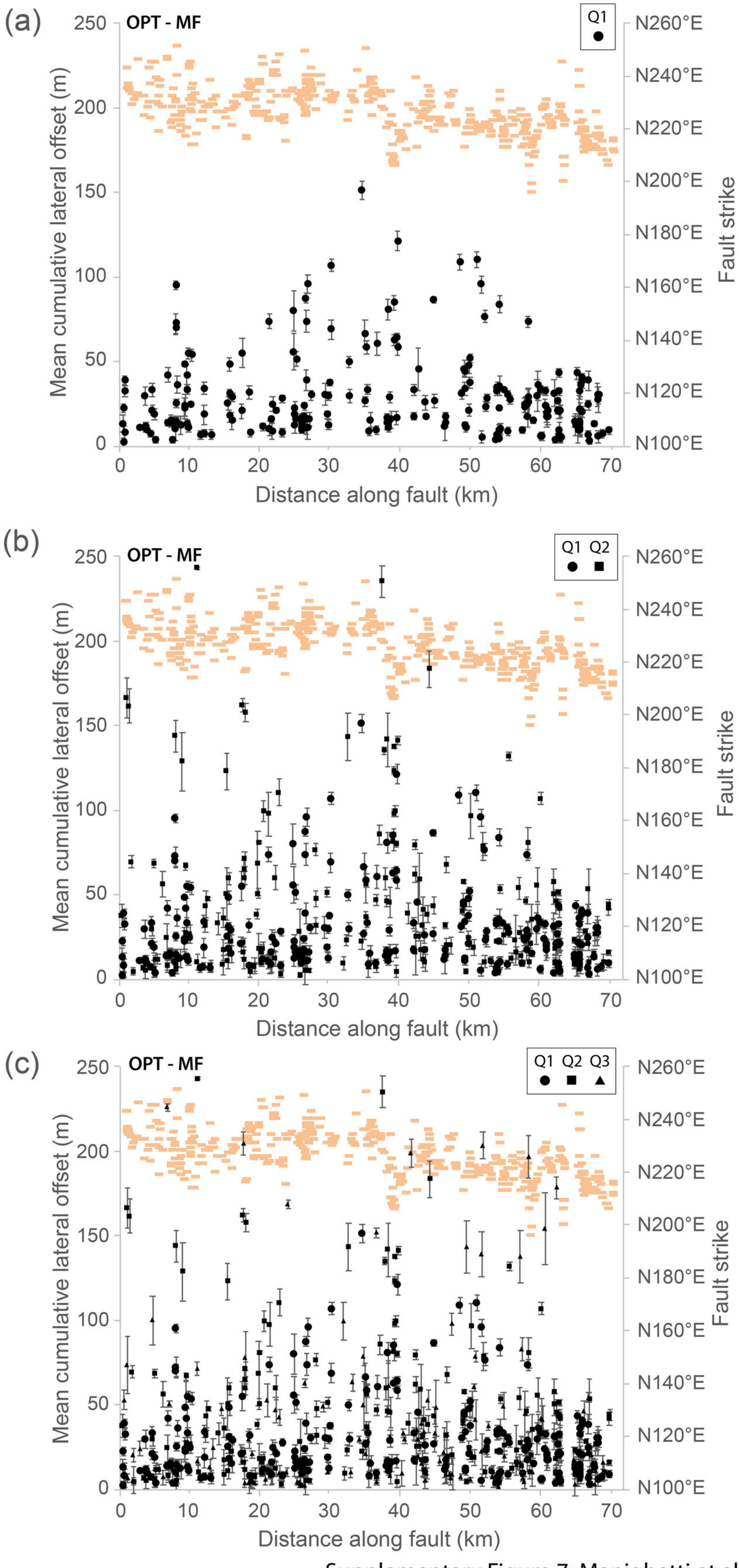

Supplementary Figure 7; Manighetti et al

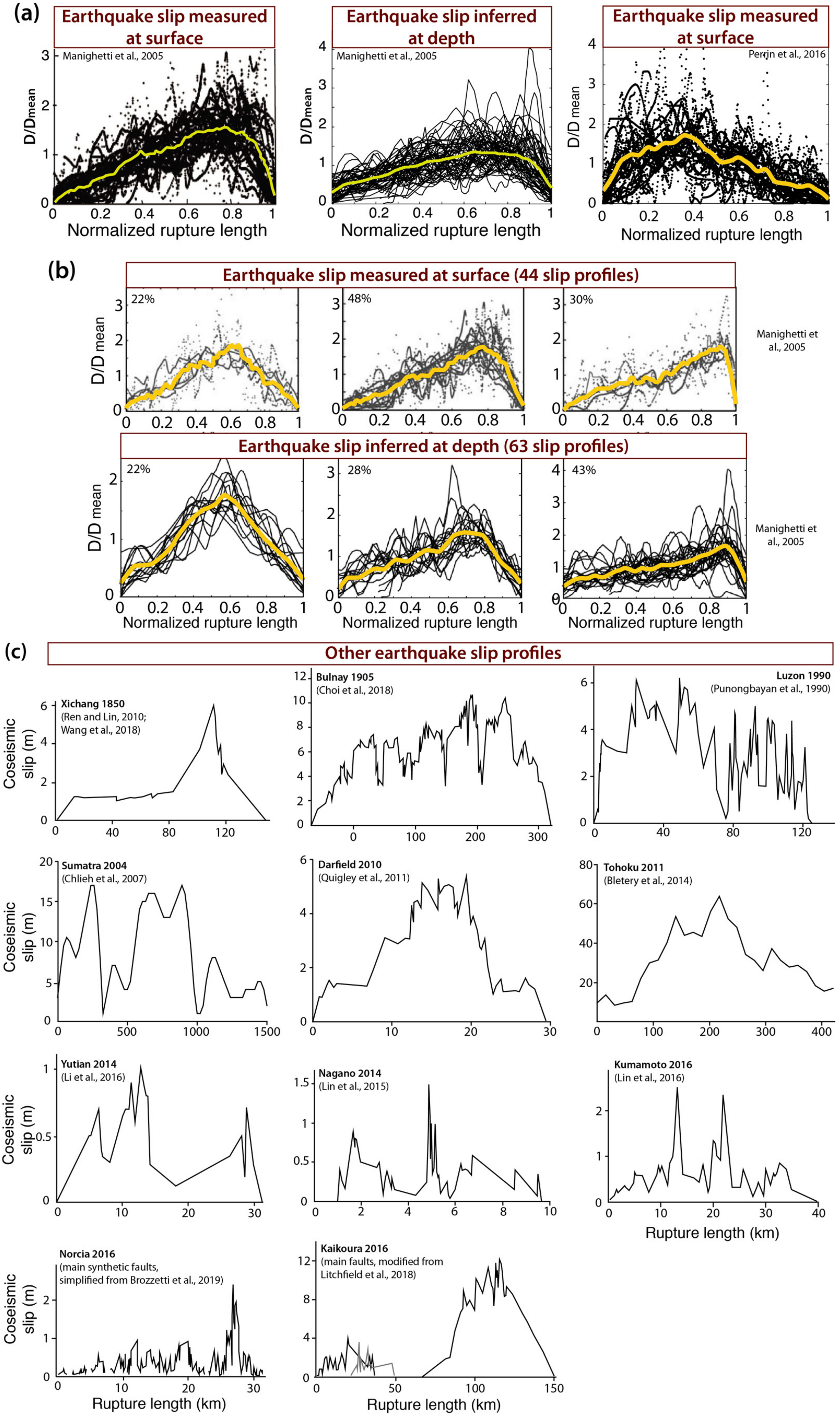

Supplementary Fig.8 - Manighetti et al.

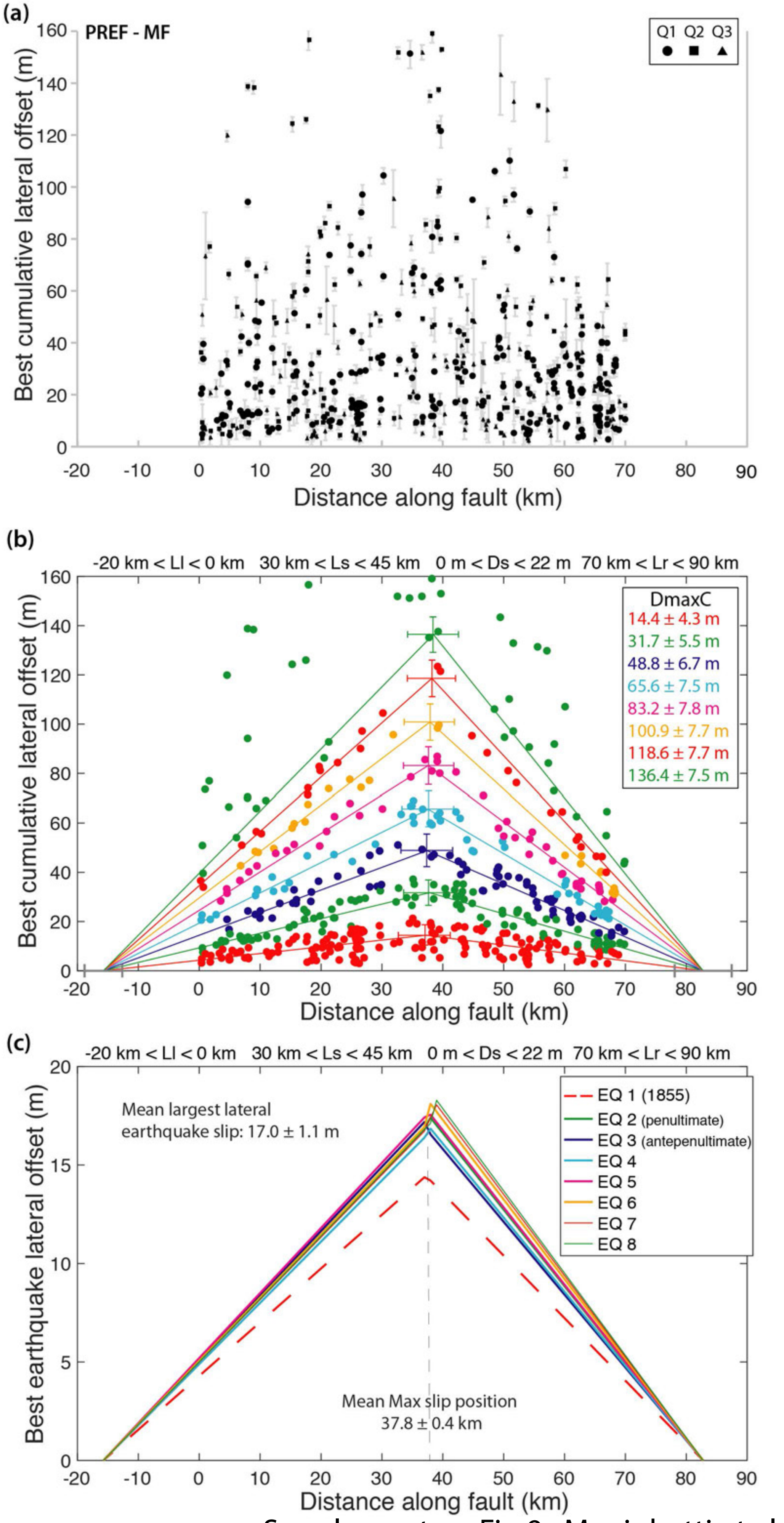

Supplementary Fig.9 ; Manighetti et al.

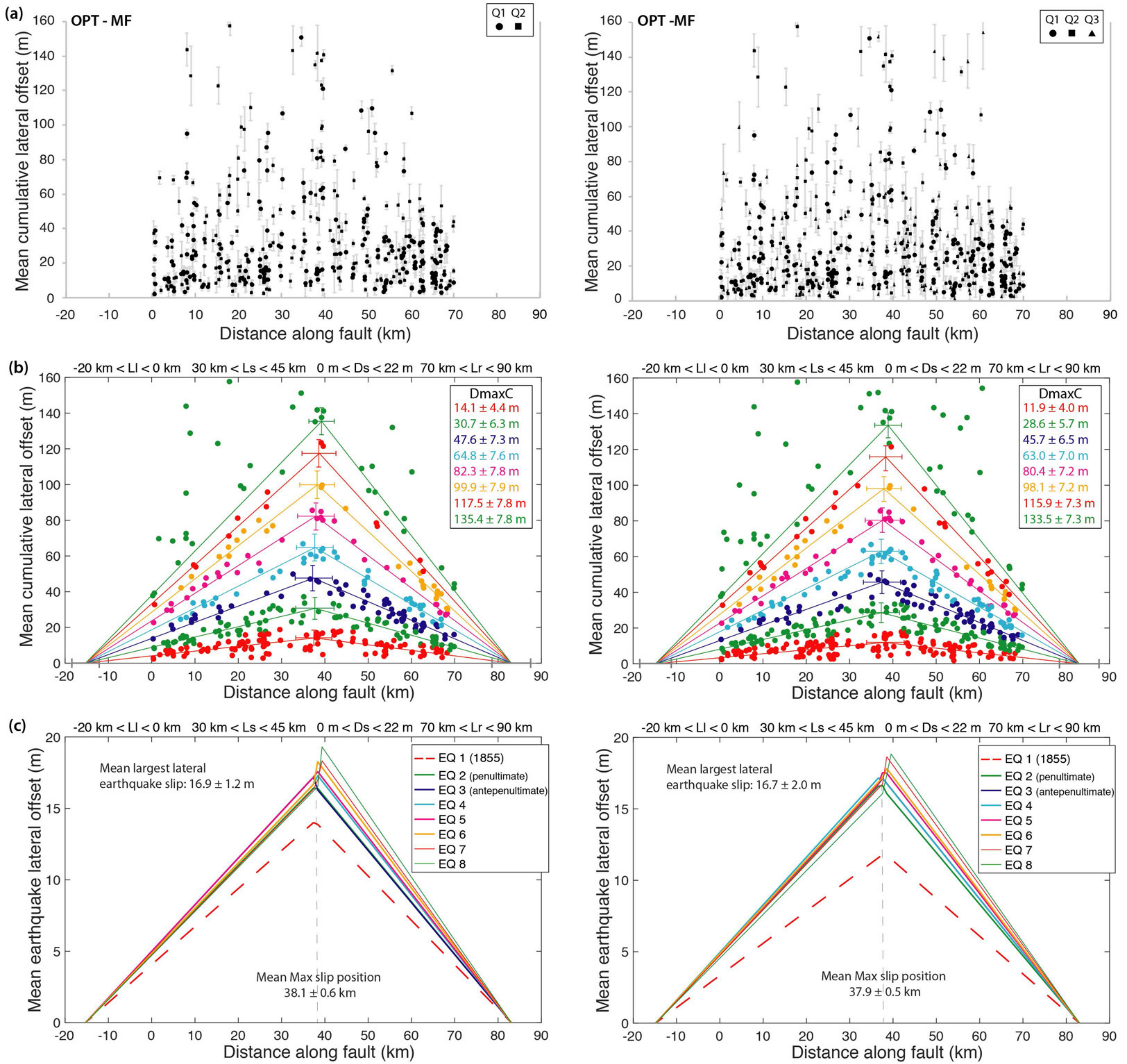

Supplementary Fig.10; Manighetti et al.

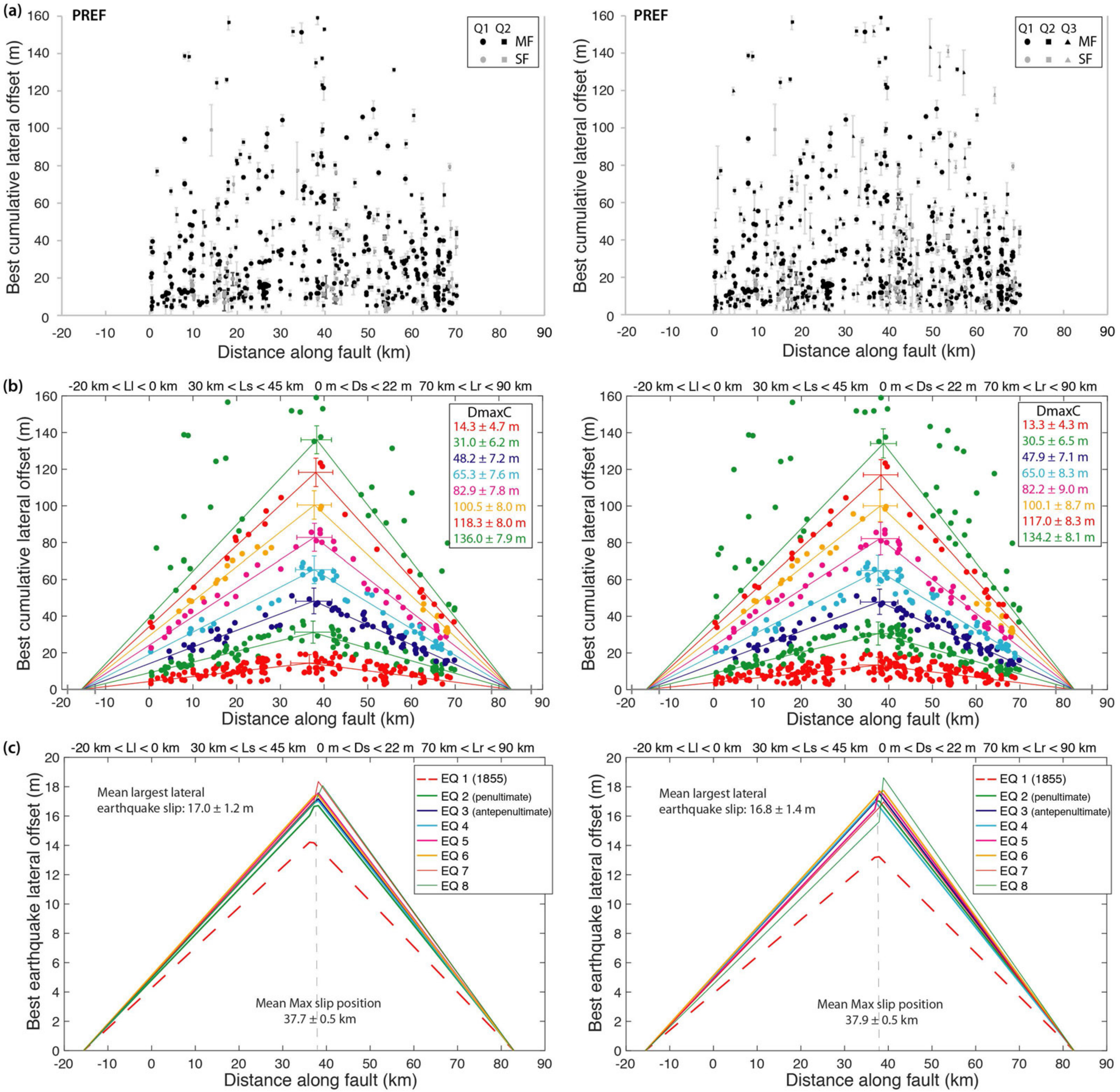

Supplementary Fig.11; Manighetti et al.

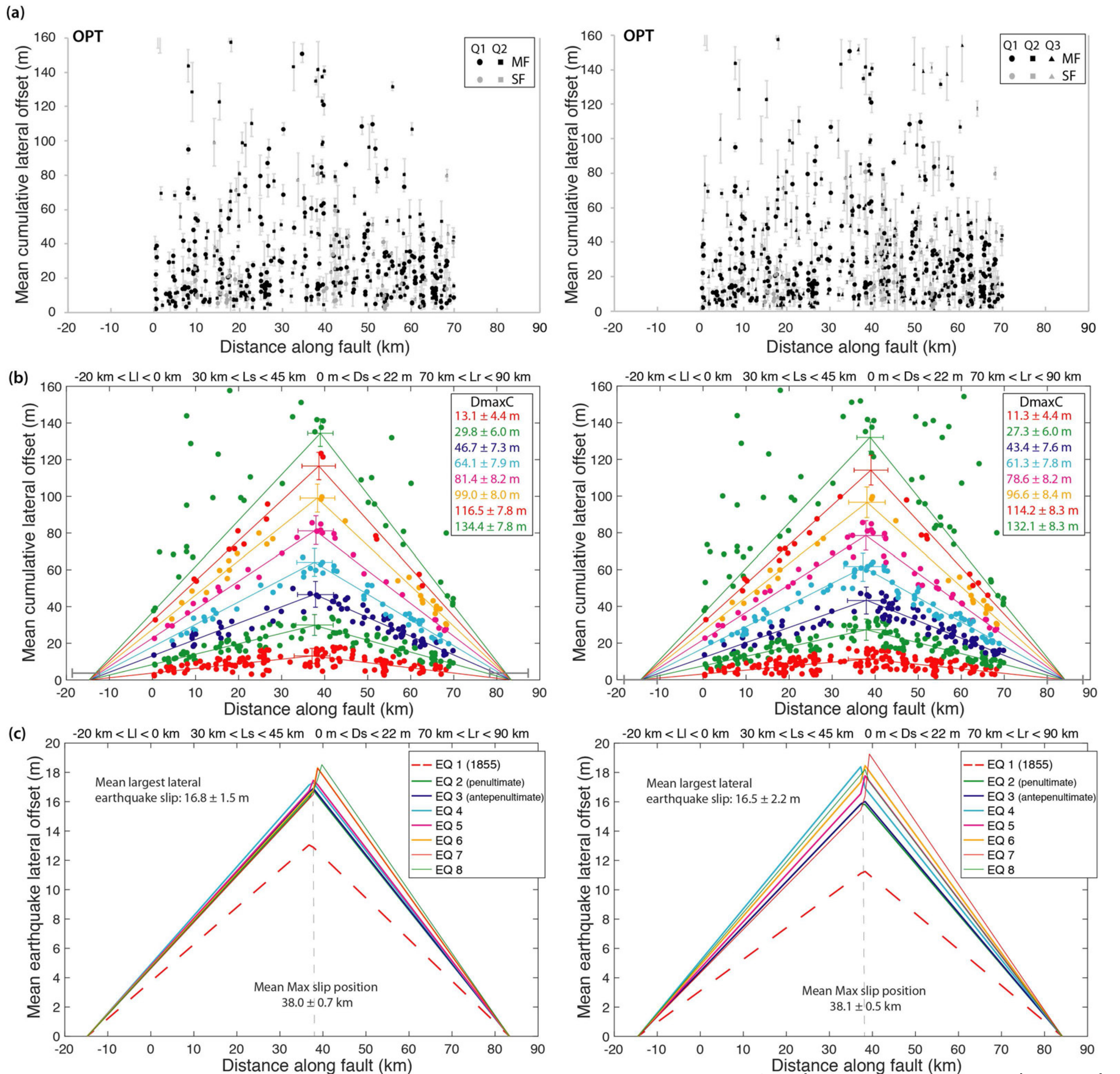

Supplementary Fig.12; Manighetti et al.

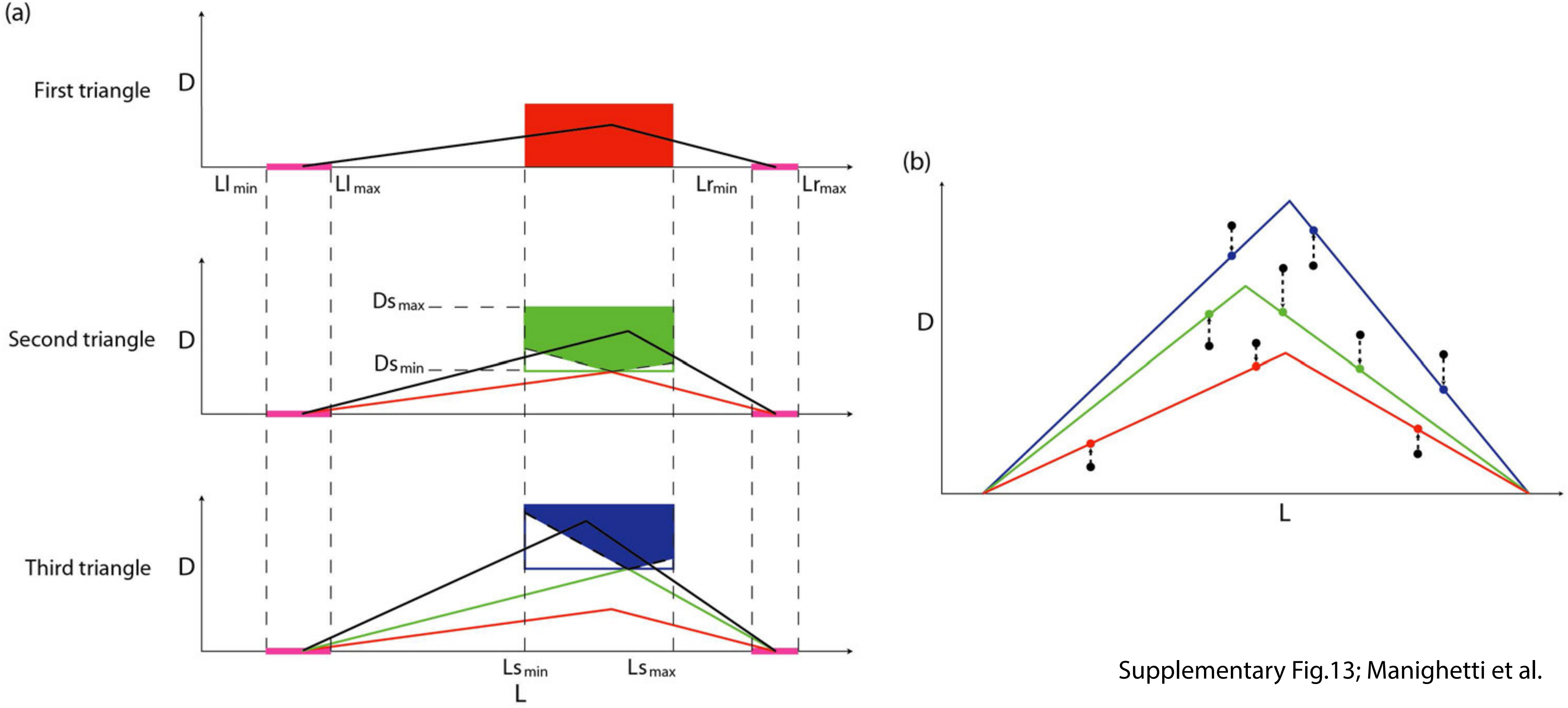

Supplementary Fig.13; Manighetti et al.

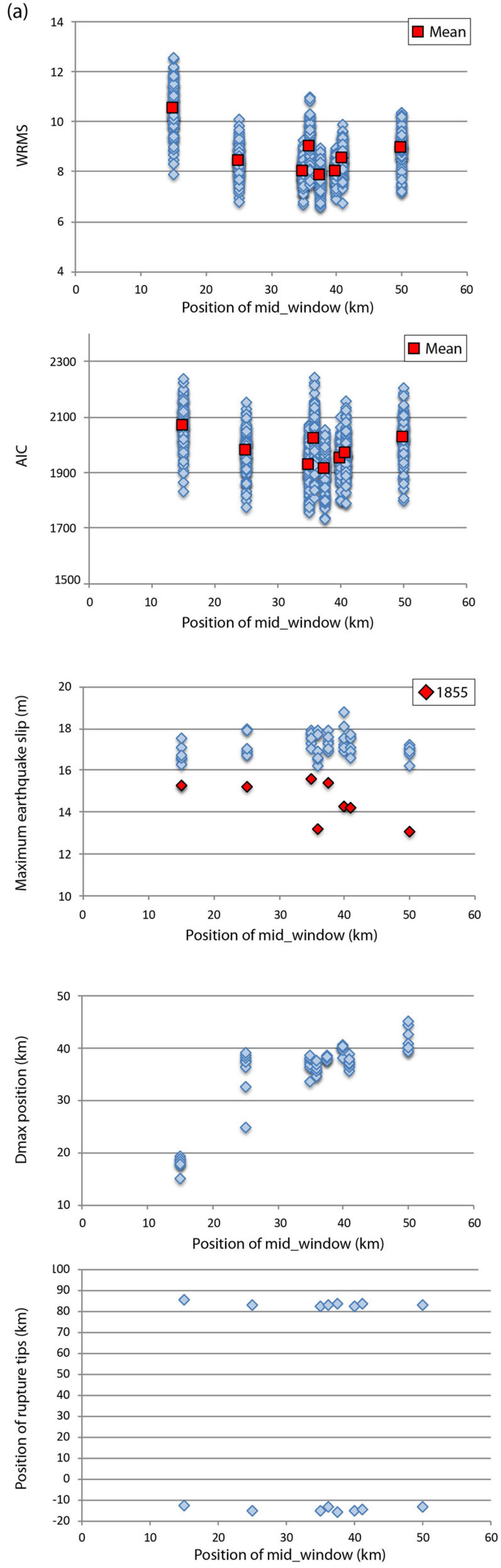

Influence of distance range for summit position search

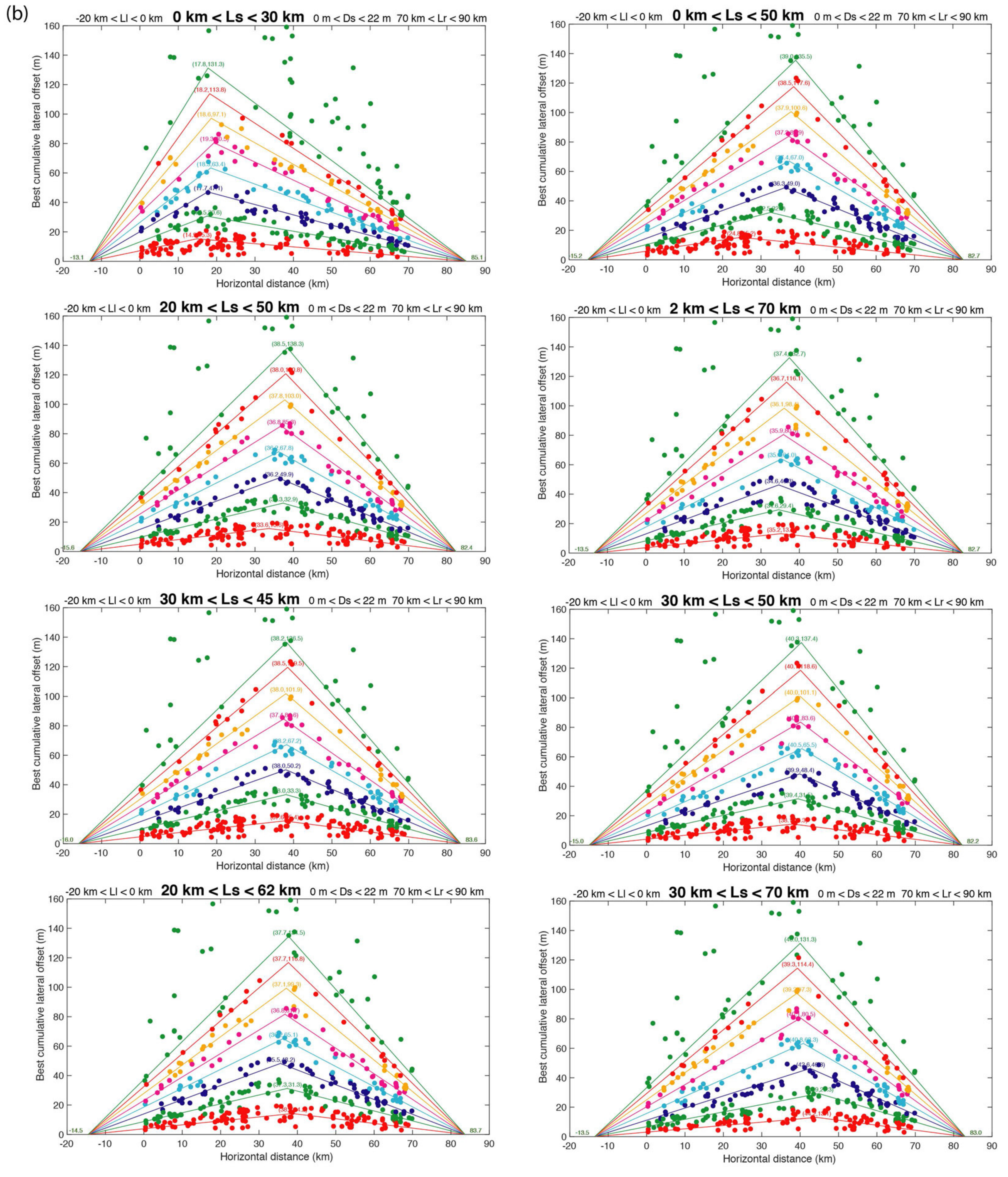

Supplementary Fig. 14A; Manighetti et al.

Influence of number of functions

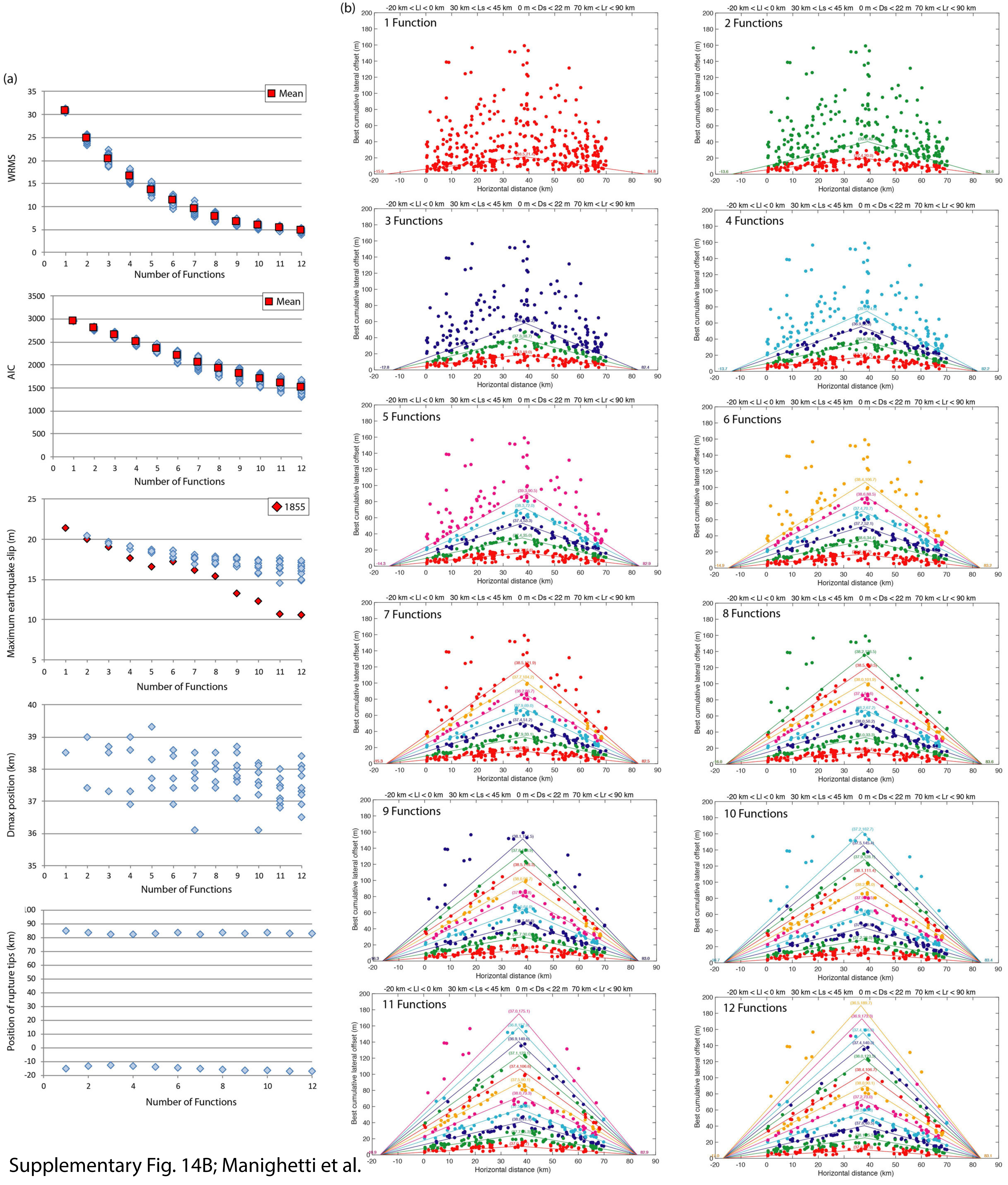

Supplementary Fig. 14B; Manighetti et al.

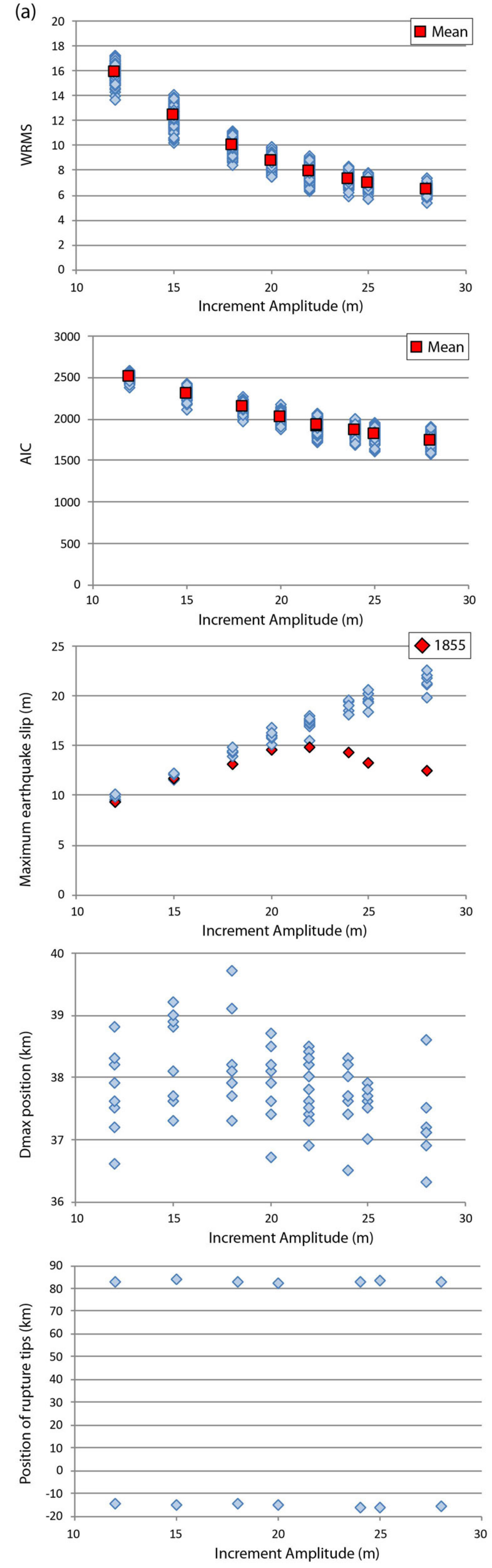

Influence of slip increment amplitude

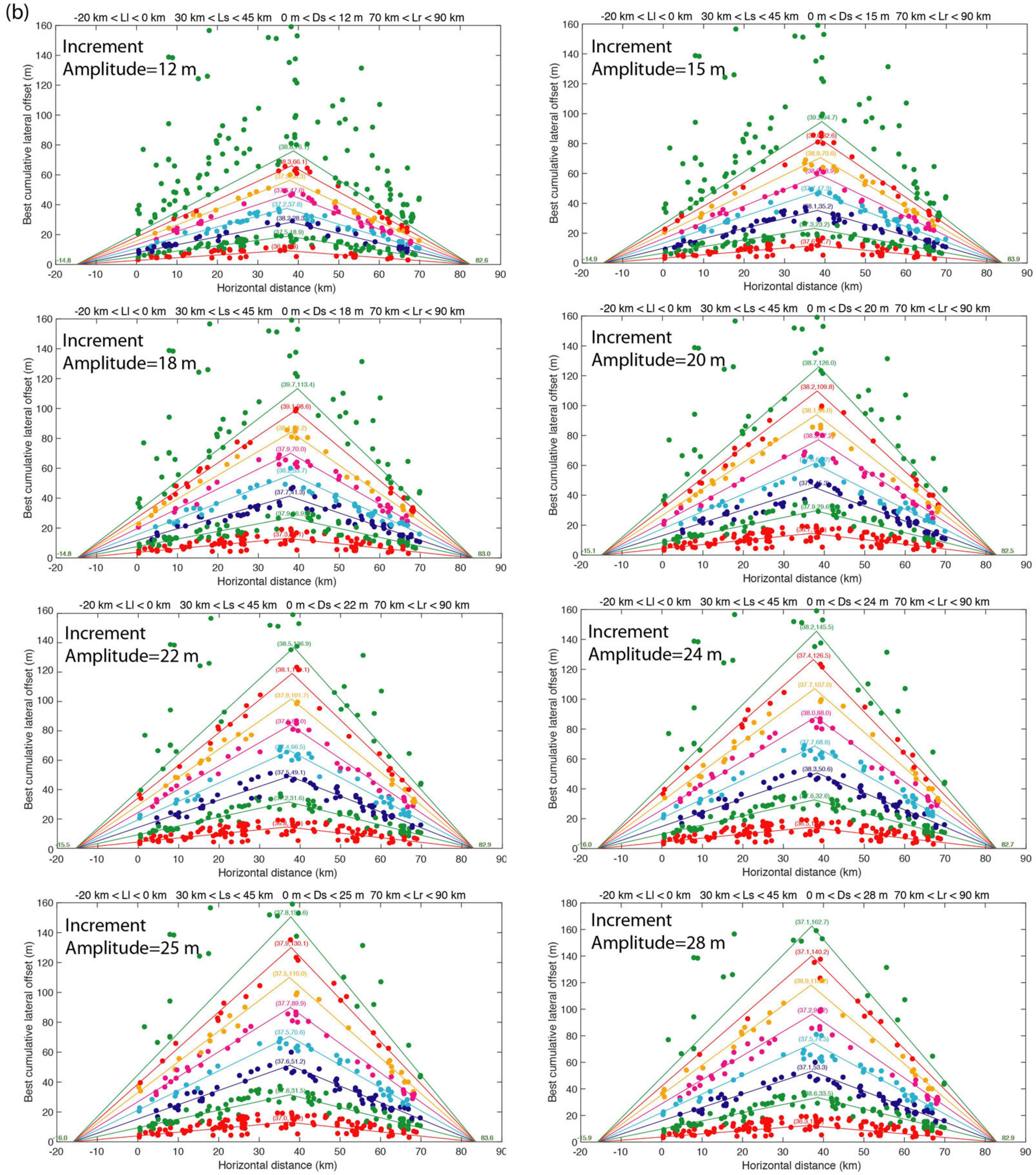

Supplementary Figure 15; Manighetti et al.

Influence of rupture length

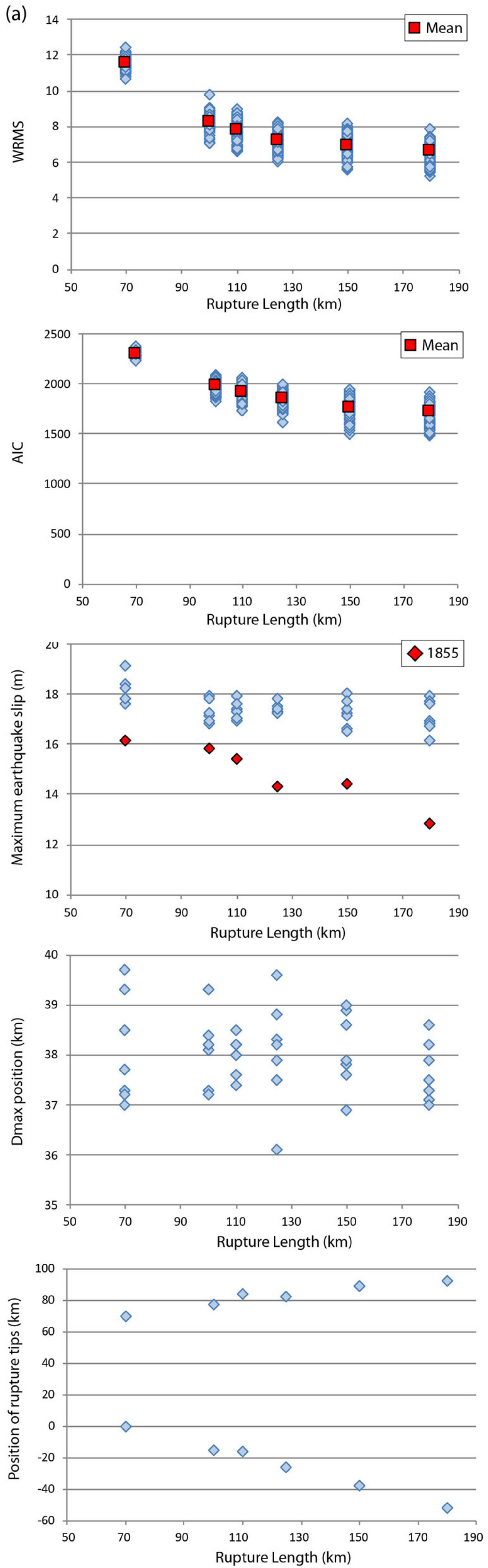

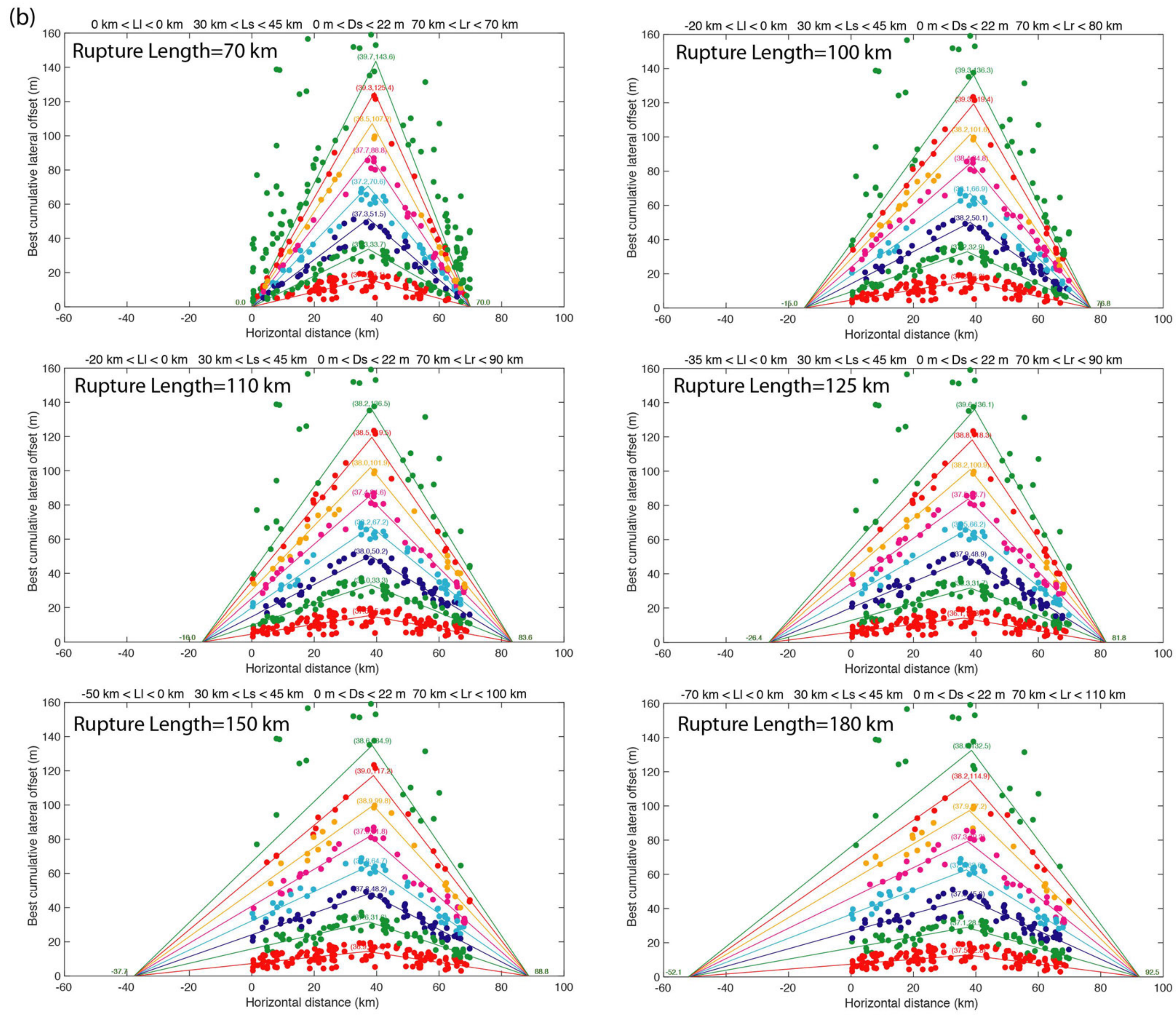

Supplementary Figure 16; Manighetti et al.

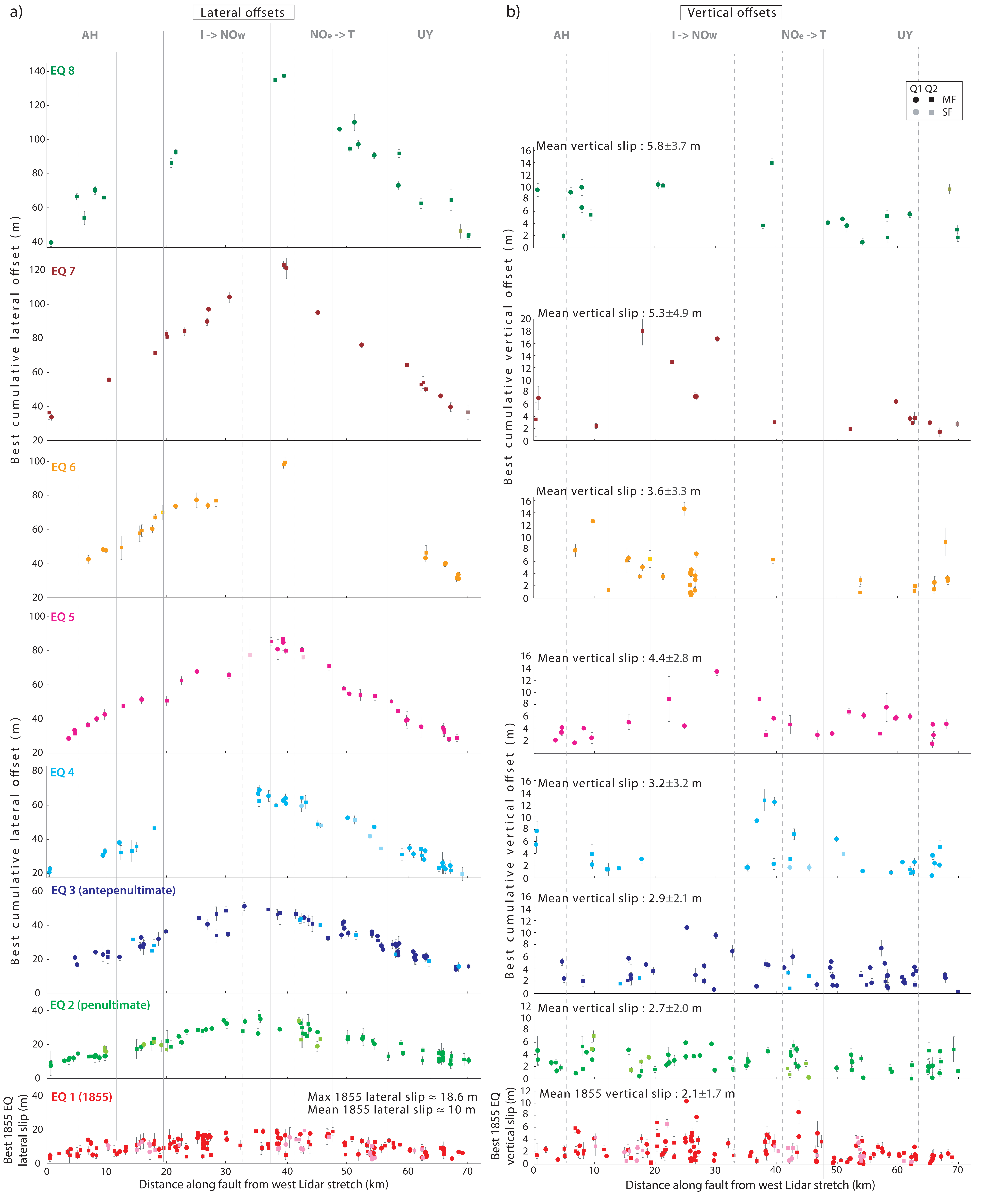

Supplementary Figure 17; Manighetti et al.

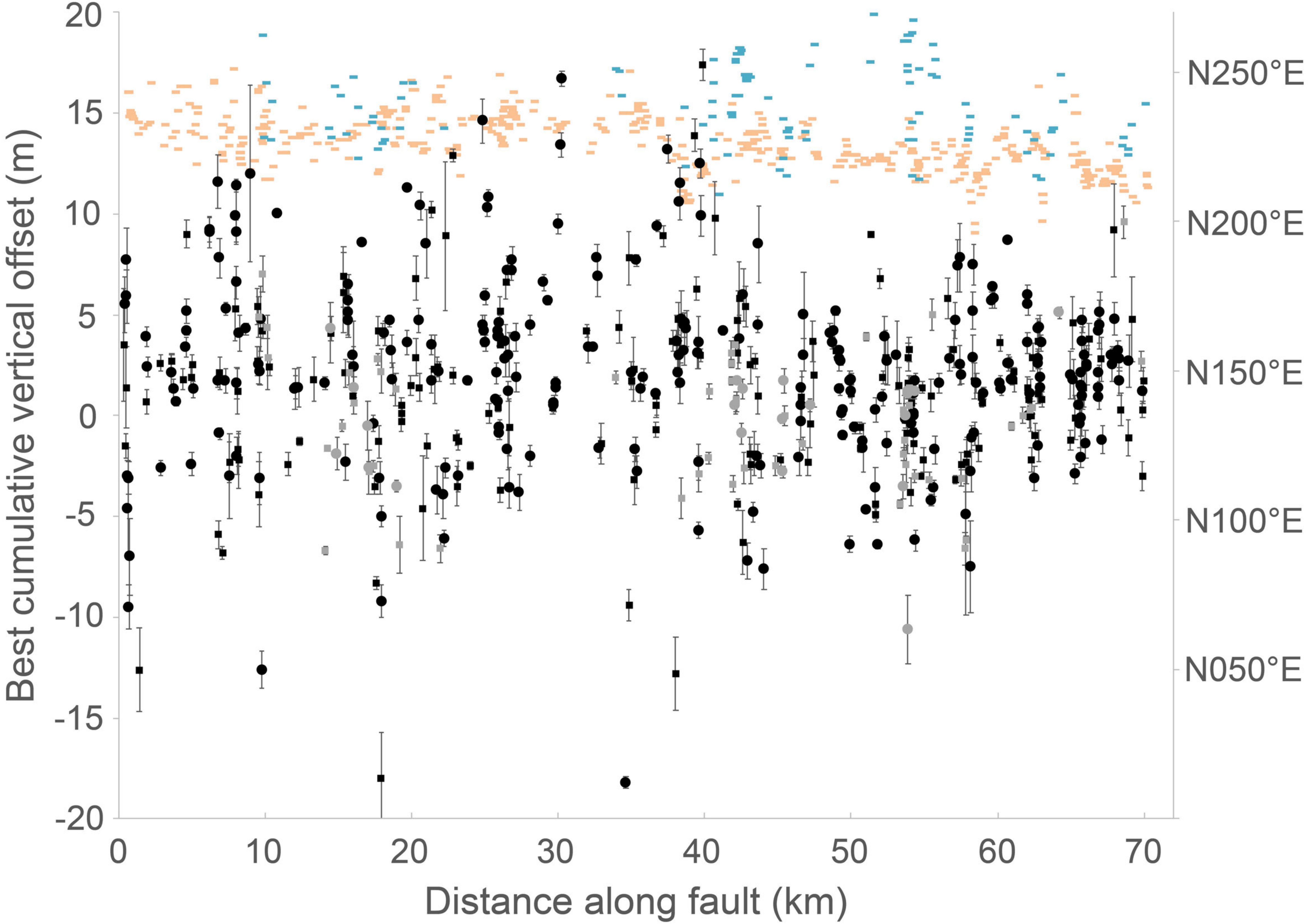

Supplementary Figure 18; Manighetti et al

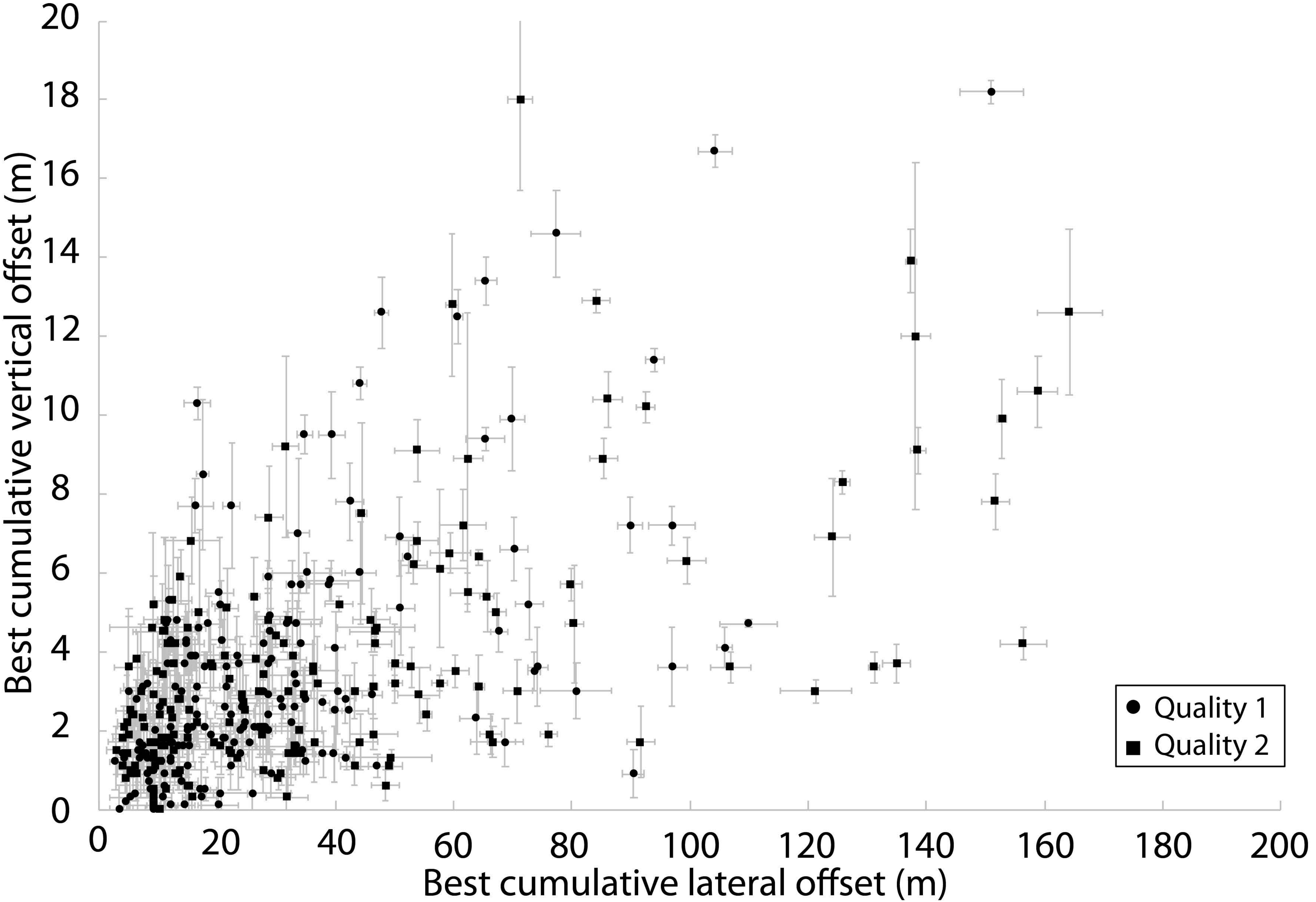

Supplementary Figure 19; Manighetti et al.

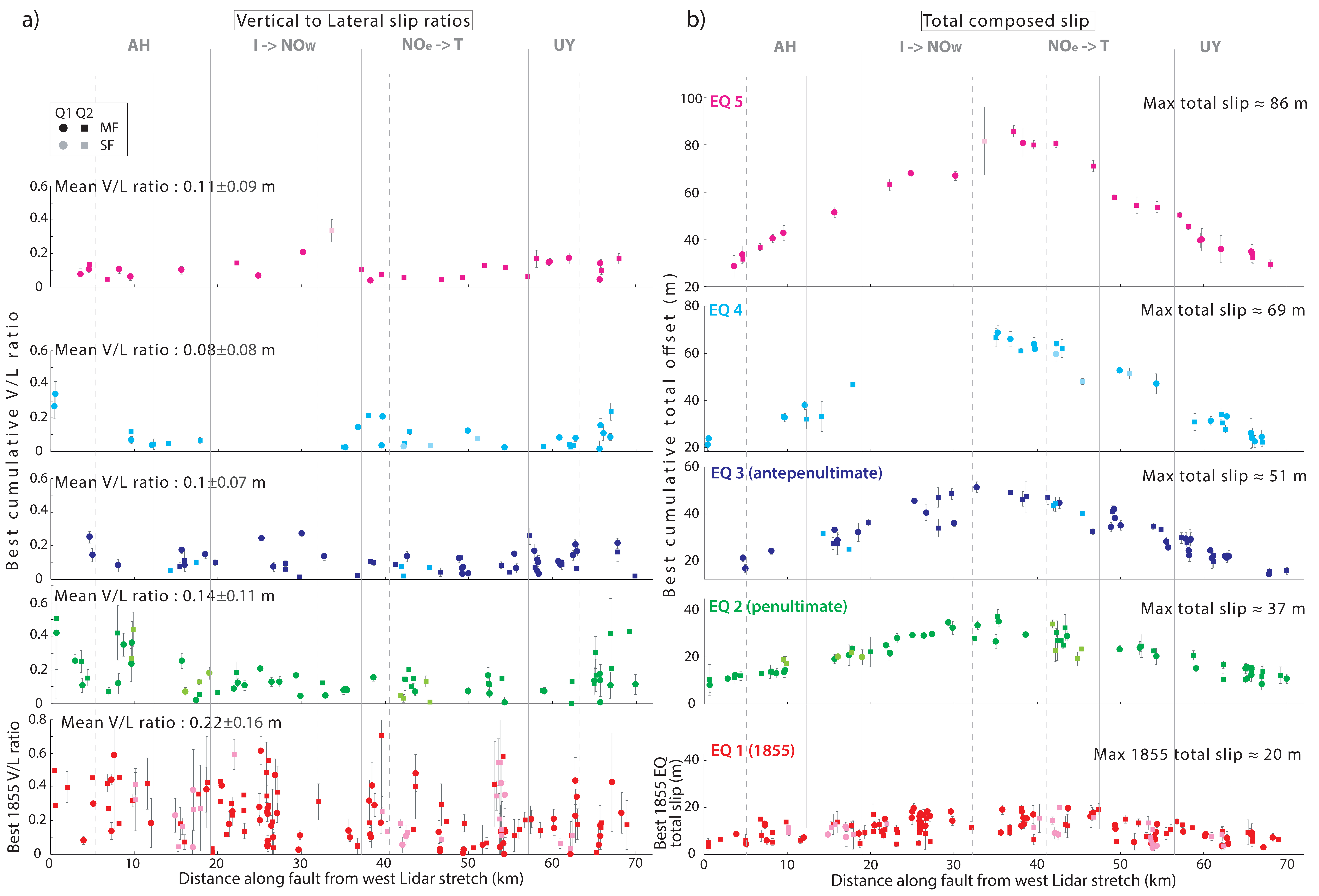

Supplementary Figure 20; Manighetti et al.

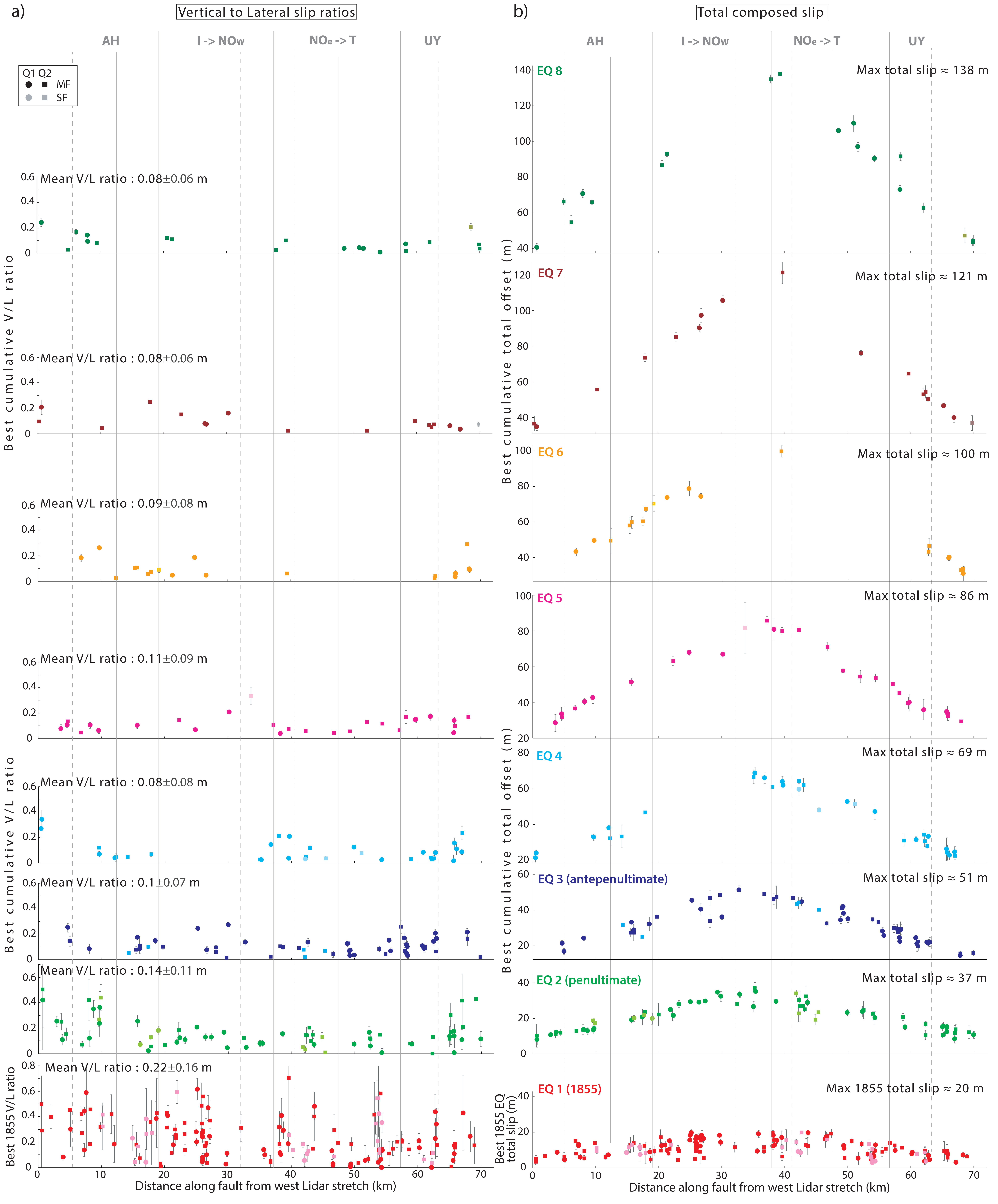

Supplementary Figure 21; Manighetti et al.

Supplementary Document A: Parameters of best lateral offset models

**Best Model PREFQ1Q2_MF (Fig. 3)**					
	Ll (km)	Lr (km)	Ls (km)	Ds (m)	Max EQ slip (m)
Function 1	-15.5 ± 3.3	82.9 ± 4.6	36.8 ± 4.4	14.6 ± 4.4	14.6
Function 2	-15.5 ± 3.3	82.9 ± 4.6	37.2 ± 4.5	31.6 ± 6.0	17
Function 3	-15.5 ± 3.3	82.9 ± 4.6	37.5 ± 4.2	49.1 ± 6.8	17.5
Function 4	-15.5 ± 3.3	82.9 ± 4.6	37.4 ± 4.3	66.5 ± 7.4	17.4
Function 5	-15.5 ± 3.3	82.9 ± 4.6	37.6 ± 4.2	84.0 ± 7.8	17.5
Function 6	-15.5 ± 3.3	82.9 ± 4.6	37.9 ± 4.2	101.7 ± 8.0	17.7
Function 7	-15.5 ± 3.3	82.9 ± 4.6	38.1 ± 3.8	119.1 ± 8.0	17.4
Function 8	-15.5 ± 3.3	82.9 ± 4.6	38.5 ± 3.5	136.9 ± 7.9	17.8
Mean	Rupt.length: 98.4 ± 5.7		37.6 ± 0.5		**17.1 ± 1.0**
	Mean WRMS : 7.81 m		Mean AICC : 1917.7		
**Best Model PREFQ1Q2Q3_MF (Supplementary Fig. 9)**					
	Ll (km)	Lr (km)	Ls (km)	Ds (m)	Max EQ slip (m)
Function 1	-15.7 ± 3.3	82.8 ± 4.8	37.2 ± 4.3	14.4 ± 4.3	14.4
Function 2	-15.7 ± 3.3	82.8 ± 4.8	37.8 ± 4.3	31.7 ± 5.5	17.3
Function 3	-15.7 ± 3.3	82.8 ± 4.8	37.4 ± 4.3	48.8 ± 6.7	17.1
Function 4	-15.7 ± 3.3	82.8 ± 4.8	37.6 ± 4.3	65.6 ± 7.5	16.8
Function 5	-15.7 ± 3.3	82.8 ± 4.8	37.6 ± 4.2	83.2 ± 7.8	17.6
Function 6	-15.7 ± 3.3	82.8 ± 4.8	37.9 ± 4.2	100.9 ± 7.7	17.7
Function 7	-15.7 ± 3.3	82.8 ± 4.8	38.2 ± 4.0	118.6 ± 7.7	17.7
Function 8	-15.7 ± 3.3	82.8 ± 4.8	38.5 ± 3.5	136.4 ± 7.5	17.8
Mean	Rupt.length: 98.5 ± 5.8		37.8 ± 0.4		**17.0 ± 1.1**
	Mean WRMS : 7.87 m		Mean AICC : 2504.2		
**Model OPTQ1Q2_MF (Supplementary Fig. 10)**					
	Ll (km)	Lr (km)	Ls (km)	Ds (m)	Max EQ slip (m)
Function 1	-15.3 ± 3.3	83.0 ± 4.6	37.7 ± 4.2	14.1 ± 4.4	14.1
Function 2	-15.3 ± 3.3	83.0 ± 4.6	37.8 ± 4.5	30.7 ± 6.3	16.3
Function 3	-15.3 ± 3.3	83.0 ± 4.6	37.4 ± 4.4	47.6 ± 7.3	16.9
Function 4	-15.3 ± 3.3	83.0 ± 4.6	37.7 ± 4.3	64.8 ± 7.6	17.2
Function 5	-15.3 ± 3.3	83.0 ± 4.6	37.9 ± 4.3	82.3 ± 7.8	17.5
Function 6	-15.3 ± 3.3	83.0 ± 4.6	38.3 ± 4.1	99.9 ± 7.9	17.6
Function 7	-15.3 ± 3.3	83.0 ± 4.6	38.7 ± 4.0	117.5 ± 7.8	17.6
Function 8	-15.3 ± 3.3	83.0 ± 4.6	39.3 ± 3.0	135.4 ± 7.8	17.9
Mean	Rupt.length: 98.3 ± 5.7		38.1 ± 0.6		**16.9 ± 1.2**
	Mean WRMS : 6.05 m		Mean AICC : 1885.7		
**Model OPTQ1Q2Q3_MF (Supplementary Fig. 10)**					
	Ll (km)	Lr (km)	Ls (km)	Ds (m)	Max EQ slip (m)
Function 1	-14.8 ± 3.8	83.0 ± 4.7	38.0 ± 4.4	11.9 ± 4.0	11.9
Function 2	-14.8 ± 3.8	83.0 ± 4.7	37.3 ± 4.3	28.6 ± 5.7	16.7
Function 3	-14.8 ± 3.8	83.0 ± 4.7	37.5 ± 4.4	45.7 ± 6.5	17.1
Function 4	-14.8 ± 3.8	83.0 ± 4.7	37.4 ± 4.2	63.0 ± 7.0	17.3
Function 5	-14.8 ± 3.8	83.0 ± 4.7	37.7 ± 4.2	80.4 ± 7.2	17.4
Function 6	-14.8 ± 3.8	83.0 ± 4.7	37.9 ± 4.0	98.1 ± 7.2	17.7
Function 7	-14.8 ± 3.8	83.0 ± 4.7	38.3 ± 3.8	115.9 ± 7.3	17.8
Function 8	-14.8 ± 3.8	83.0 ± 4.7	38.9 ± 3.2	133.5 ± 7.3	17.6
Mean	Rupt.length: 97.8 ± 6.0		37.9 ± 0.5		**16.7 ± 2.0**
	Mean WRMS : 6.08 m		Mean AICC : 2558.8		

Model PREFQ1Q2_MF+SF (Supplementary Fig. 11)					
	Ll (km)	Lr (km)	Ls (km)	Ds (m)	Max EQ slip (m)
Function 1	-15.5 ± 3.3	82.9 ± 4.7	36.6 ± 4.3	14.3 ± 4.7	14.3
Function 2	-15.5 ± 3.3	82.9 ± 4.7	37.5 ± 4.3	31.0 ± 6.2	16.7
Function 3	-15.5 ± 3.3	82.9 ± 4.7	37.7 ± 4.2	48.2 ± 7.2	17.2
Function 4	-15.5 ± 3.3	82.9 ± 4.7	37.8 ± 4.3	65.3 ± 7.6	17.1
Function 5	-15.5 ± 3.3	82.9 ± 4.7	37.8 ± 4.1	82.9 ± 7.8	17.6
Function 6	-15.5 ± 3.3	82.9 ± 4.7	37.8 ± 4.0	100.5 ± 8.0	17.6
Function 7	-15.5 ± 3.3	82.9 ± 4.7	38.1 ± 4.0	118.3 ± 8.0	17.8
Function 8	-15.5 ± 3.3	82.9 ± 4.7	38.3 ± 3.5	136.0 ± 7.9	17.7
Mean	Rupt.length: 98.4 ± 5.7		37.7 ± 0.5		**17.0 ± 1.2**
	Mean WRMS : 7.43 m		Mean AICC : 2187.2		
**Model PREFQ1Q2Q3_MF+SF (Supplementary Fig. 11)**					
	Ll (km)	Lr (km)	Ls (km)	Ds (m)	Max EQ slip (m)
Function 1	-15.6 ± 3.3	82.5 ± 4.3	37.5 ± 4.5	13.3 ± 4.3	13.3
Function 2	-15.6 ± 3.3	82.5 ± 4.3	37.5 ± 4.2	30.5 ± 6.5	17.2
Function 3	-15.6 ± 3.3	82.5 ± 4.3	37.8 ± 4.4	47.9 ± 7.1	17.4
Function 4	-15.6 ± 3.3	82.5 ± 4.3	37.6 ± 4.0	65.0 ± 8.3	17.1
Function 5	-15.6 ± 3.3	82.5 ± 4.3	38.0 ± 4.4	82.2 ± 9.0	17.2
Function 6	-15.6 ± 3.3	82.5 ± 4.3	38.1 ± 4.0	100.1 ± 8.7	17.9
Function 7	-15.6 ± 3.3	82.5 ± 4.3	38.2 ± 4.0	117.0 ± 8.3	16.9
Function 8	-15.6 ± 3.3	82.5 ± 4.3	38.8 ± 3.0	134.2 ± 8.1	17.2
Mean	Rupt.length: 98.1 ± 5.4		37.9 ± 0.5		**16.8 ± 1.4**
	Mean WRMS : 7.55 m		Mean AICC : 3053.1		
**Model OPTQ1Q2_MF+SF (Supplementary Fig. 12)**					
	Ll (km)	Lr (km)	Ls (km)	Ds (m)	Max EQ slip (m)
Function 1	-14.8 ± 3.8	83.4 ± 4.3	37.0 ± 4.3	13.1 ± 4.4	13.1
Function 2	-14.8 ± 3.8	83.4 ± 4.3	37.6 ± 4.4	29.8 ± 6.0	16.7
Function 3	-14.8 ± 3.8	83.4 ± 4.3	37.6 ± 4.3	46.7 ± 7.3	16.9
Function 4	-14.8 ± 3.8	83.4 ± 4.3	37.6 ± 4.2	64.1 ± 7.9	17.4
Function 5	-14.8 ± 3.8	83.4 ± 4.3	37.8 ± 4.2	81.4 ± 8.2	17.3
Function 6	-14.8 ± 3.8	83.4 ± 4.3	38.3 ± 4.1	99.0 ± 8.0	17.6
Function 7	-14.8 ± 3.8	83.4 ± 4.3	38.7 ± 3.9	116.5 ± 7.8	17.5
Function 8	-14.8 ± 3.8	83.4 ± 4.3	39.0 ± 3.0	134.4 ± 7.8	17.9
Mean	Rupt.length: 98.2 ± 5.7		38.0 ± 0.7		**16.8 ± 1.5**
	Mean WRMS : 5.89 m		Mean AICC : 2162.2		
**Model OPTQ1Q2Q3_MF+SF (Supplementary Fig. 12)**					
	Ll (km)	Lr (km)	Ls (km)	Ds (m)	Max EQ slip (m)
Function 1	-14.5 ± 4.0	84.1 ± 4.3	38.1 ± 4.6	11.3 ± 4.4	11.3
Function 2	-14.5 ± 4.0	84.1 ± 4.3	37.9 ± 4.5	27.3 ± 6.0	16.0
Function 3	-14.5 ± 4.0	84.1 ± 4.3	38.0 ± 4.4	43.4 ± 7.6	16.1
Function 4	-14.5 ± 4.0	84.1 ± 4.3	37.4 ± 4.5	61.3 ± 7.8	17.9
Function 5	-14.5 ± 4.0	84.1 ± 4.3	37.8 ± 3.9	78.6 ± 8.2	17.3
Function 6	-14.5 ± 4.0	84.1 ± 4.3	38.1 ± 4.3	96.6 ± 8.4	18.0
Function 7	-14.5 ± 4.0	84.1 ± 4.3	39.0 ± 4.0	114.2 ± 8.3	17.6
Function 8	-14.5 ± 4.0	84.1 ± 4.3	38.8 ± 3.1	132.1 ± 8.3	17.9
Mean	Rupt.length: 98.6 ± 5.9		38.1 ± 0.5		**16.5 ± 2.2**
	Mean WRMS : 6.14 m		Mean AICC : 3089.2		
**GLOBAL MEAN**	**Rupture length: 98.3 ± 5.7 km**		**Mean Ls: 37.9 ± 0.5 km**		**Max EQ slip: 16.9 ± 1.4 m**

Figures where models are shown are indicated in brackets.

The best models are the average models calculated by the outermost routine from the multiple Nr "best models" derived from the intermediate routine (see Methods). Here we generally chose Nr=500, and therefore each of the best models is derived from 50 000 iterations.

Ll is left (west) distance of rupture western tip with respect to main fault western end in Lidar data, taken as 0 km. Lr is right (east) distance of rupture eastern tip with respect to main fault eastern end in Lidar data, which is 70 km. Ls is position of largest lateral slip along the [0-70 km] main fault in Lidar data. Ds is largest modeled lateral slip. In all cases, error is standard deviation of the Nr models. Errors on Ds are conservative and overestimated because their calculation is not weighted with the quality of the Nr models (i.e., a weaker model with larger WRMS and AICC parameters has same weight as a more robust model with smaller WRMS and AICC).

"Max EQ slip" is largest earthquake lateral slip derived from Ds values. For reasons explained above, we do not translate the Ds errors on the Max EQ slips.

Mean values of parameters are provided for each model, along with the mean WRMS and AICC criteria of the model.

Best Model PREFQ1Q2_MF is calculated from quality 1 and quality 2 PREF lateral offsets (in range 0-160m) measured across the main fault. Data are weighted by both their uncertainty and quality weight. The input parameters for the best model calculation are M_N_fit_rand_tri13(500,100,L,D,W,8,-20,0,30,45,0,22,70,90), that is : Nr=500, Nt=100, 8 functions are searched, range for western rupture tip (with respect to western end of Lidar stretch) is [-20-0 km], range for along-fault search of largest slip is [30-45 km], range for earthquake slip search is [0-22 m], and range for eastern rupture tip (with respect to eastern end of Lidar stretch) is [70-90 km]. These input parameter ranges are the same for the other models below.

Best Model PREFQ1Q2Q3_MF is calculated from quality 1, quality 2 and quality 3 PREF lateral offsets (in range 0-160m) measured across the main fault. Data are weighted by both their uncertainty and quality weight. The input parameters for the best model calculation are M_N_fit_rand_tri13(500,100,L,D,W,8,-20,0,30,45,0,22,70,90) (as above).

Best Model OPTQ1Q2_MF is calculated from quality 1 and quality 2 OPT lateral offsets (in range 0-160m) measured across the main fault. Data are weighted by both their uncertainty and quality weight. The input parameters for the best model calculation are M_N_fit_rand_tri13(500,100,L,D,W,8,-20,0,30,45,0,22,70,90) (as above).

Best Model OPTQ1Q2Q3_MF is calculated from quality 1, quality 2, and quality 3 OPT lateral offsets (in range 0-160m) measured across the main fault. Data are weighted by both their uncertainty and quality weight. The input parameters for the best model calculation are M_N_fit_rand_tri13(500,100,L,D,W,8,-20,0,30,45,0,22,70,90) (as above).

Best Model PREFQ1Q2_MF+SF is calculated from quality 1 and quality 2 PREF lateral offsets (in range 0-160m) measured across both the main fault and the secondary faults. Data are weighted by both their uncertainty and quality weight. Input parameters for the model calculation are M_N_fit_rand_tri13(500,100,L,D,W,8,-20,0,30,45,0,22,70,90) (as above).

Best Model PREFQ1Q2Q3_MF+SF is calculated from quality 1, quality 2 and quality 3 PREF lateral offsets (in range 0-160m) measured across both the main fault and the secondary faults. Data are weighted by both their uncertainty and quality weight. Input parameters for the model calculation are M_N_fit_rand_tri13(100,100,L,D,W,8,-20,0,30,45,0,22,70,90) (as above but with Nr=100).

Best Model OPTQ1Q2_MF+SF is calculated from quality 1 and quality 2 OPT lateral offsets (in range 0-160m) measured across both the main fault and the secondary faults. Data are weighted by both their uncertainty and quality weight. Input parameters for the model calculation are M_N_fit_rand_tri13(500,100,L,D,W,8,-20,0,30,45,0,22,70,90) (as above).

Best Model OPTQ1Q2Q3_MF+SF is calculated from quality 1, quality 2 and quality 3 OPT lateral offsets (in range 0-160m) measured across both the main fault and the secondary faults. Data are weighted by both their uncertainty and quality weight. Input parameters for model calculation are M_N_fit_rand_tri13(100,100,L,D,W,8,-20,0,30,45,0,22,70,90) (as above but with Nr=100).

Global means in red are average from all model means, calculated with Gaussian probability density functions.
